# The anti-tumor drug 2-hydroxyoleic acid (Minerval) stimulates signaling and retrograde transport

**DOI:** 10.18632/oncotarget.13508

**Published:** 2016-11-22

**Authors:** Maria L. Torgersen, Tove Irene Klokk, Simona Kavaliauskiene, Christian Klose, Kai Simons, Tore Skotland, Kirsten Sandvig

**Affiliations:** ^1^ Department of Molecular Cell Biology, Institute for Cancer Research, The Norwegian Radium Hospital, Oslo University Hospital, Norway, and Centre for Cancer Biomedicine, Faculty of Medicine, University of Oslo, Norway; ^2^ Department of Molecular Biosciences, University of Oslo, Norway; ^3^ Lipotype GmbH, Dresden, Germany

**Keywords:** hydroxyoleic acid, minerval, membrane lipid therapy, EGFR, ricin

## Abstract

2-hydroxyoleic acid (OHOA, Minerval^®^) is an example of a substance used for membrane lipid therapy, where the cellular membranes rather than specific proteins constitute the therapeutical target. OHOA is thought to mediate its anti-tumor effect by affecting the biophysical properties of membranes, which leads to altered recruitment and activation of amphitropic proteins, altered cellular signaling, and eventual cell death. Little is known about the initial signaling events upon treatment with OHOA, and whether the altered membrane properties would have any impact on the dynamic intracellular transport system. In the present study we demonstrate that treatment with OHOA led to a rapid release of intracellular calcium and activation of multiple signaling pathways in HeLa cells, including the PI3K-AKT1-MTOR pathway and several MAP kinases, in a process independent of the EGFR. By lipidomics we confirmed that OHOA was incorporated into several lipid classes. Concomitantly, OHOA potently increased retrograde transport of the plant toxin ricin from endosomes to the Golgi and further to the endoplasmic reticulum. The OHOA-stimulated ricin transport seemed to require several amphitropic proteins, including Src, phospholipase C, protein kinase C, and also Ca^2+^/calmodulin. Interestingly, OHOA induced a slight increase in endosomal localization of the retromer component VPS35. Thus, our data show that addition of a lipid known to alter membrane properties not only affects signaling, but also intracellular transport.

## INTRODUCTION

In the search for novel therapeutical options in cancer treatment it has become increasingly clear that in addition to targeting specific proteins, the cellular membrane constitutes an attractive target. This treatment option, termed membrane lipid therapy, is based on addition of rationally designed lipids to alter membrane composition and structure, which in turn would affect the localization and activity of key membrane associated proteins, and their downstream events [1, 2]. This concept has partly emerged from the observed health effects of the Mediterranean diet rich in olive oil. The therapeutic effect of oleic acid (OA), the predominant fatty acid in olive oil, is however limited due to its use as fuel through β-oxidation in the mitochondria. In contrast, the 2-hydroxylated, synthetic analog of OA (OHOA) is believed to have a slower metabolism, a longer half-life, and thus a more long-lasting pharmacological effect [1]. An increased half-life due to slight modifications of lipid drugs is known from other antineoplastic agents, such as edelfosine, which is provided with increased stability via a methylether-modification of its lyso-phosphatidylcholine backbone [3]. OHOA, which is registered under the name Minerval^®^, is one of the best studied examples of membrane lipid therapy and is currently in phase I/II clinical trials for glioma (ClinicalTrials.gov Identifier NCT01792310) [4].

The biophysical properties of a membrane not only regulate the activity of proteins embedded in it, as reviewed in [5], but also the recruitment and activity of peripheral, amphitropic membrane proteins that bind weakly and reversibly to membrane lipids and are only temporarily associated to the membrane. The membrane recruitment of amphitropic proteins is mediated via various hydrophobic anchor structures, such as lipid binding pockets, amphipathic helices, hydrophobic loops, or post-translationally lipidated amino acid residues [6]. The activity of several key signaling proteins, including Ras, Src family kinases, phospholipase C (PLC), protein kinase C (PKC), and G-proteins, is regulated by amphitropism [6, 7].

Minerval^®^ is thought to mediate its anti-tumor effect via alterations in amphitropic mechanisms [1]. OHOA has been shown to spontaneously incorporate into the membrane bilayer of model membranes, to alter the membrane microdomain organization, and increase membrane fluidity and hydration [2, 8–12]. Moreover, OHOA has been shown to destabilize the lamellar phase and increase the propensity of model membranes to form hexagonal or other non-lamellar structures [4, 11]. These lipid packing alterations are mainly thought to affect the activity and membrane recruitment of G-proteins, PKC, or adenylyl cyclase [1, 2]. Prolonged OHOA treatment has been associated with induction of ER stress, autophagy, and cell cycle arrest [13, 14]. OHOA has also been reported to activate sphingomyelin synthase and restore the levels of sphingomyelin (SM) to normal levels in glioma cells [14, 15].

Although several long-term effects of OHOA have been reported, the initial cellular response to treatment with this fatty acid is less studied, and any potential effect on membrane trafficking is unknown. In this study we wanted to elucidate the early effects of OHOA on cellular signaling and membrane dynamics. To study membrane flow in drug-treated cells we used the protein toxin ricin as a probe. Ricin can be regarded as a membrane marker as it binds to both glycoproteins and glycolipids exhibiting a terminal galactose [16]. Upon internalization the majority of toxin molecules are either recycled to the cell surface or targeted for degradation in the endolysosomal pathway. In addition, a small fraction of ricin molecules follows the retrograde pathway from endosomes to the *trans*-Golgi network (TGN) and further to the endoplasmic reticulum (ER). From the ER the toxic moiety is translocated to the cytosol to inhibit protein synthesis. Thus, ricin probes the major cellular transport pathways, and is a convenient test molecule to determine alterations in membrane trafficking events. In addition, retrograde transport of ricin is regulated by proteins that might be sensitive to alterations in membrane fluidity or curvature, such as members of the sorting nexin (SNX) family [17] and dynamin [18].

Our data reveal that treatment with OHOA increases membrane fluidity and rapidly stimulates several signaling pathways in HeLa cells. Concomitantly, OHOA potently increases the retrograde transport of ricin to the Golgi in a process that seems to require calmodulin and signaling through amphitropic kinases.

## RESULTS

### Treatment with OHOA leads to increased membrane fluidity and release of intracellular Ca^2+^

Addition of free fatty acids to membranes is known to change several biophysical parameters, such as curvature, fluidity, phase behavior, permeability, and microdomain organization [5, 12]. These effects occur either as a result of incorporation of the free fatty acid into the membrane, or altered fatty acid composition of phospholipids. As the main therapeutical effect of OHOA is proposed to be alterations in membrane properties, and OHOA has been reported to increase membrane fluidity in model membranes [8–10, 12], we wanted to know whether changes in lipid packing are detectable in the plasma membrane of OHOA-treated HeLa cells. To this end, we used the environment-sensitive probe NR12S, which is reported to detect changes selectively in the outer leaflet of the plasma membrane. Indeed, a weak increase in membrane fluidity was observed after 30 minutes of OHOA treatment (Supplementary Figure S1). This indicates that the added OHOA directly interacts with the surface of HeLa cells.

**Figure 1:**
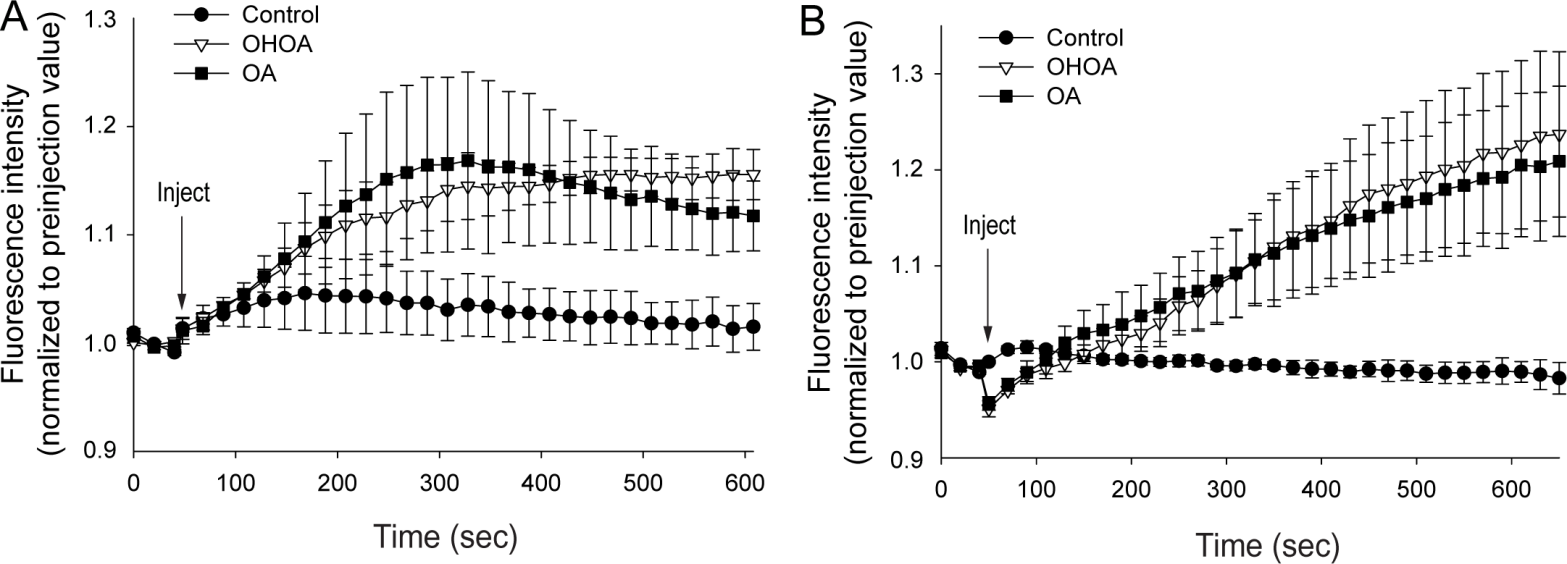
Both OHOA and OA stimulate release of calcium from intracellular stores HeLa cells were loaded with cell-permeable Fluo-4 in a buffer with calcium (**A**) or without calcium (**B**), followed by addition of 25 μM OHOA or OA (or ethanol as control). The fluorescence intensity was measured every 20 seconds for 10 minutes, and the data were normalized to the level of fluorescence before addition of the compound. The mean values of three independent experiments are shown ± SEM.

G-proteins and PKC are reported as the main targets of OHOA treatment downstream of the alterations in membrane properties [1]. As these targets are both associated with Ca^2+^ signaling, we tested whether OHOA would increase the levels of cytosolic calcium, [Ca^2+^]_i_. The unmodified OA was included for comparison. Indeed, OHOA rapidly increased [Ca^2+^]_i_ during the first 10 minutes of incubation, and OA had a very similar effect during this short-term experiment (Figure 1A). The observed increase in [Ca^2+^]_i_ might be explained via two different scenarios; either the altered membrane properties lead to opening of plasma membrane calcium channels, or alternatively, it leads to activation of membrane-associated proteins and downstream signaling events involving the second messengers such as diacylglycerol (DAG) and inositol trisphosphate (IP_3_), and subsequent release of Ca^2+^ from intracellular stores, such as the ER. To explore these possibilities, the Ca^2+^ measurements were repeated in Ca^2+^-free buffer. Interestingly, the same rapid increase was observed in Ca^2+^-free buffer (Figure 1B), suggesting that both OHOA and OA promote cellular signaling and Ca^2+^ release from intracellular stores.

### OHOA activates multiple signaling pathways

**Figure 2:**
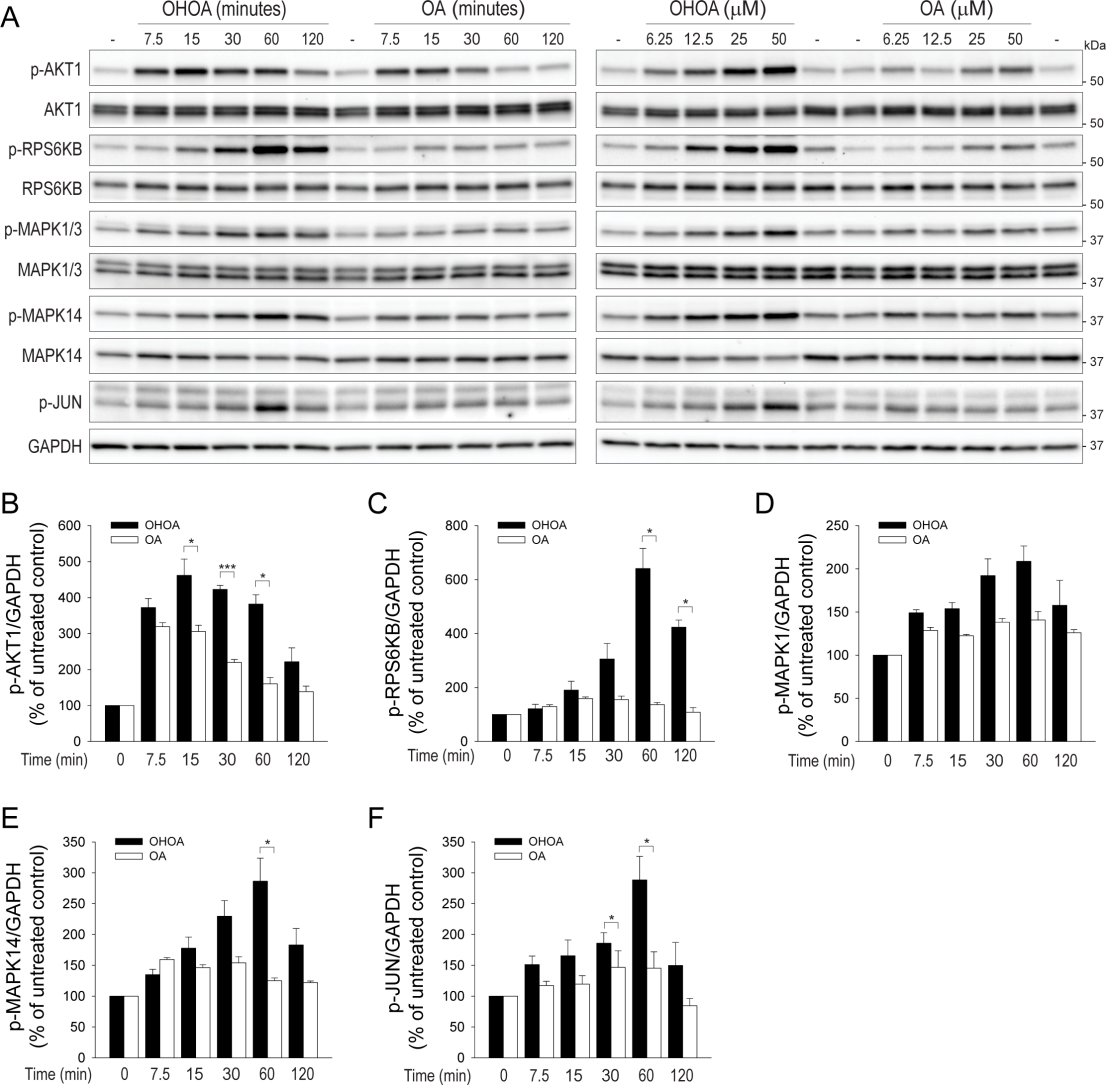
OHOA stimulates cellular signaling (**A**) HeLa cells were treated with 12.5 μM OHOA or OA for the indicated time periods (left panel) or treated for 30 minutes with the indicated concentrations of OHOA or OA (right panel) and cell lysates were prepared for immunoblotting. The blots were probed with the indicated antibodies, and representative blots are shown. The relative levels of p-AKT1 (**B**), p-RPS6KB (**C**), p-MAPK1 (**D**), p-MAPK14 (**E**), and p-JUN (**F**) were normalized to GAPDH. All bars show mean values + SEM quantified from at least three independent experiments; *p < 0.05; ***p < 0.001.

Next, we wanted to study the fatty acid-induced intracellular signaling in more detail. The PI3K pathway is downstream of both G-proteins and receptor tyrosine kinases, and a typical readout for PI3K activity is the phosphorylation of AKT1. Indeed, both OA and OHOA rapidly induced phosphorylation of AKT1, peaking within 15 minutes, however, the OHOA-induced signal was significantly higher (Figure 2A, 2B). Interestingly, whereas the OA-induced phosphorylation of AKT1 was transient and returned almost to basal levels within 1 hour, OHOA-induced AKT1 phosphorylation remained elevated for at least 1 hour before the signal declined (Figure 2B). Thus, it seems that OHOA is able to induce a more potent and sustained cellular stimulation than OA. To see whether the differential stimulation of PI3K affects downstream signaling in the PI3K-AKT1-MTOR pathway, we probed for phosphorylation of p70-S6K/RPS6KB, a well-known substrate of MTOR. Whereas OA only stimulated MTOR at the highest concentrations applied, OHOA strongly stimulated phosphorylation of RPS6KB both in a time- and concentration-dependent manner, peaking at 60 minutes after addition (Figure 2A, 2C; Supplementary Figure S2A). Furthermore, to see whether other signaling pathways were differentially affected by the two fatty acids, we probed for phosphorylation of the MAP kinases ERK/MAPK1, p38/MAPK14 and JNK/MAPK8. All the tested signaling pathways were more potently activated by OHOA than by OA both in a time- and concentration-dependent manner with maximal activation after 1 hour (Figure 2D–2FD; Supplementary Figure S2B–S2D). Together, this indicates that low concentrations of OHOA are able to induce sustained signaling in these cells, whereas higher concentrations of OA are required. OHOA has previously been reported to induce ER stress upon long-term treatment of glioma cells [13]. However, ER stress was not induced by OHOA in HeLa cells within the 2 hour time-frame relevant for this study (Supplementary Figure S2E).

### OHOA does not activate the EGFR or ERBB2

**Figure 3:**
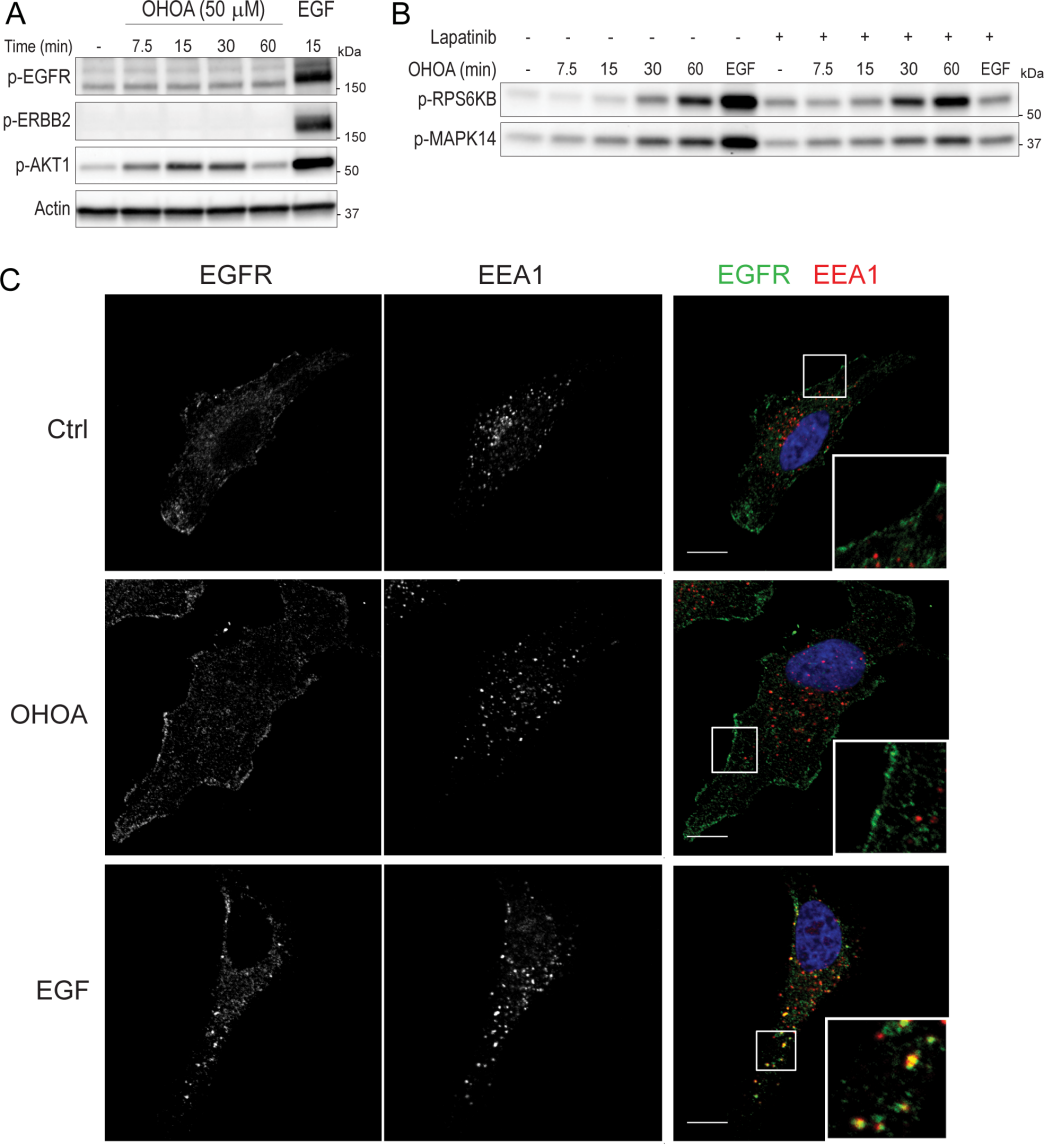
OHOA does not activate or internalize the EGFR (**A**) HeLa cells were treated with 50 μM OHOA for the indicated time periods and cell lysates were prepared for immunoblotting. Treatment with 20 ng/ml EGF for 15 minutes was used as a positive control. The blots were probed with the indicated antibodies. (**B**) HeLa cells were pretreated with 2 μM lapatinib for 30 minutes before addition of 12.5 μM OHOA or 20 ng/ml EGF for 15 minutes, and cell lysates were prepared for immunoblotting. The blots were probed with the indicated antibodies. (**C**) HeLa cells were treated with 25 μM OHOA or 20 ng/ml EGF for 15 minutes, fixed and prepared for immunofluorescence with the indicated antibodies. Scale bar; 10 μm.

OA has previously been shown to activate the EGFR in a ligand-independent manner [19, 20], and also to downregulate the expression of ERBB2 in breast cancer cells [21]. We therefore wanted to know whether OHOA has the ability to activate and/or internalize members of the EGFR family in HeLa cells. As before, treatment with OHOA led to phosphorylation of AKT1, however, neither the EGFR nor ERBB2 was phosphorylated by OHOA stimulation (Figure 3A). In line with this, OHOA-induced phosphorylation of RPS6KB and MAPK14 were not blocked by the dual EGFR/ERBB2 inhibitor lapatinib, whereas EGF-induced signaling was totally abolished (Figure 3B). Moreover, OHOA did not stimulate internalization of the EGFR, whereas EGF stimulated a rapid uptake of the receptor (Figure 3C), and degradation of ^125^I-EGF was virtually unaltered by treatment with OHOA (Supplementary Figure S3). Together, this indicates that OHOA-induced signaling is not downstream of the EGFR/ERBB2.

### OHOA stimulates ricin toxicity without affecting endocytosis, recycling or degradation

**Figure 4:**
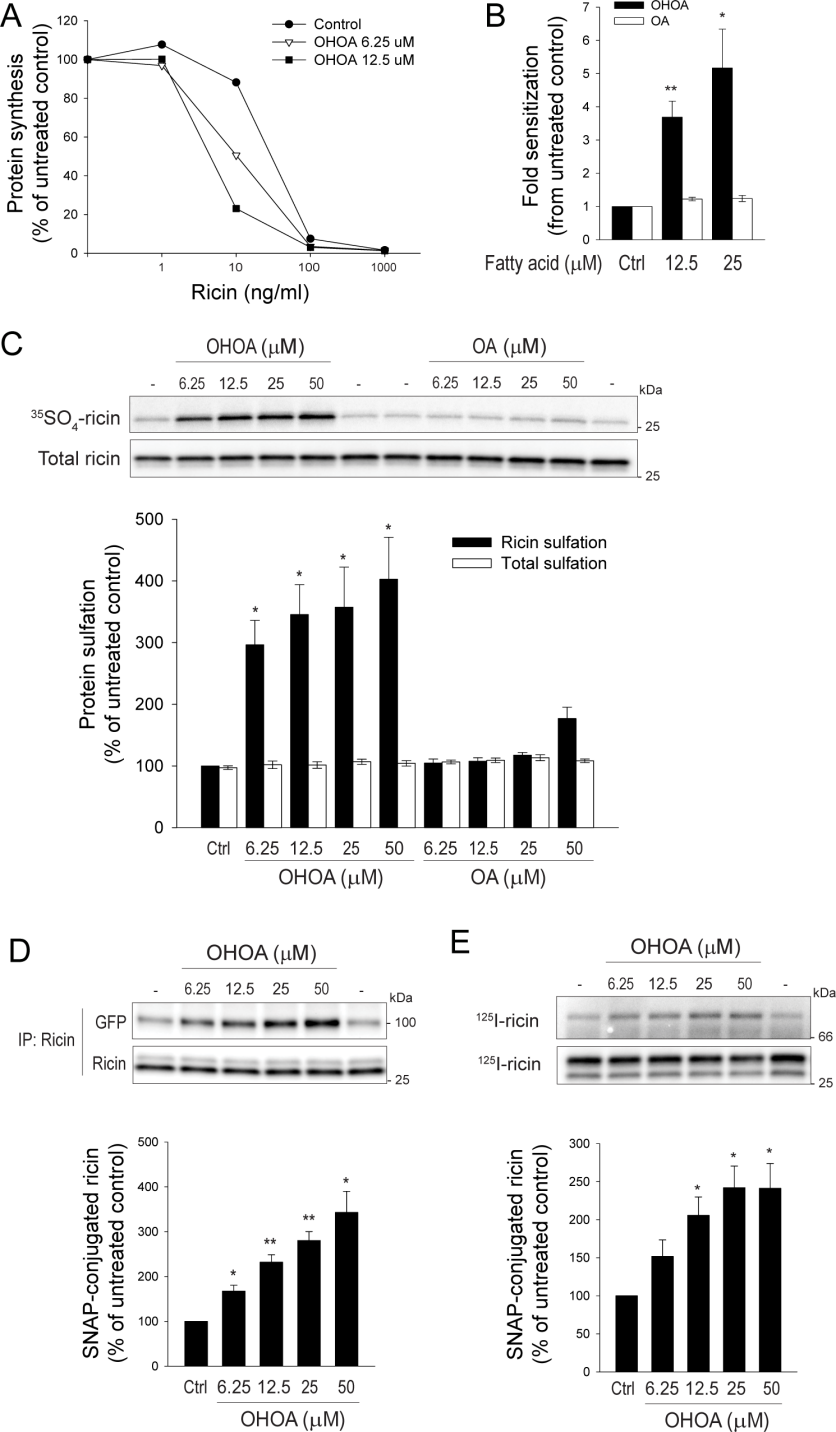
OHOA stimulates ricin toxicity and retrograde transport (**A**–**B**). HeLa cells were preincubated with the indicated concentrations of OHOA or OA in leucine-free medium for 30 minutes, then increasing concentrations of ricin were added and the incubation continued for 3 hours. The protein synthesis was measured as described in Materials and Methods. (A) Data from one representative experiment. (B) The bars represent fold sensitization to ricin at 50% inhibition of protein synthesis. (**C**) HeLa cells were treated with the indicated concentrations of OHOA or OA and ricin sulfation was determined as described in Materials and Methods. A representative sulfation autoradiograph with the corresponding immunoblot is shown, and band intensities were quantified. (**D**) HeLa-GalT-GFP-SNAP cells were treated with the indicated concentrations of OHOA and Golgi transport of ricin was determined by the SNAP-tag assay as described in Materials and Methods. Representative immunoblots of total- and SNAP-tagged ricin are shown, and band intensities were quantified. (**E**) HeLa-ER-mCherry-SNAP cells were treated with the indicated concentrations of OHOA, and ER transport of ricin was determined by the SNAP-tag assay as described in Materials and Methods. Representative autoradiographs of total- and SNAP-tagged ricin are shown, and band intensities were quantified. All bars show mean values ± SEM quantified from at least 3 independent experiments; *p < 0.05; **p < 0.01 compared to untreated control.

The biophysical properties of membranes do not only regulate key signaling pathways, but also the dynamic intracellular membrane transport system [22]. Importantly, these processes are intertwined, as membrane transport might influence the amplitude of growth factor signaling by for instance internalization of activated growth factor receptors, regulated recycling back to the cell surface, or termination of the signal by sorting the receptors for lysosomal degradation [23, 24]. Although OHOA did not affect internalization or degradation of the EGFR, the possibility exists that OHOA-induced alterations of membrane properties and cellular signaling would affect other intracellular transport pathways. As a probe for cellular membrane flow we used the plant toxin ricin, and first measured the end-point of ricin transport, the protein synthesis inhibition caused by the enzymatic subunit of the toxin. Interestingly, ricin toxicity was strongly potentiated in HeLa cells upon treatment with OHOA, whereas OA had limited effect (Figure 4A, 4B). This stimulatory effect of OHOA was not specific to HeLa cells, as increased ricin toxicity was observed in the two other cell lines tested, U2-OS and HEp-2 cells (Supplementary Figure S4).

Increased ricin toxicity might be caused by a higher accessibility to ricin molecules, or an alteration in the itinerary of the available toxin molecules. The amount of internalized ricin was not increased by OHOA (Supplementary Figure S5A), suggesting that the increased toxicity is not merely caused by a higher uptake of ricin molecules. Although ricin intoxication strictly depends on routing of toxin molecules from endosomes into the retrograde pathway, this constitutes only a minor fraction of the total internalized ricin molecules. The majority is either recycled to the plasma membrane or sorted into the lysosomal pathway for degradation. Interestingly, drugs such as bafilomycin A1 or chloroquine that inhibit either recycling or degradation of ricin, and thus lead to a net accumulation of intracellular ricin, have been shown to increase ricin toxicity [25]. However, ricin recycling was not reduced by OHOA (Supplementary Figure S5B), and the degradation of ricin was only marginally reduced by OHOA compared to the full inhibition upon treatment with bafilomycin A1 or concanamycin A (Supplementary Figure S5C), suggesting that sorting into the retrograde pathway is selectively promoted by OHOA.

### OHOA stimulates transport of ricin to the Golgi and the ER

To verify that sorting into the retrograde pathway was stimulated by OHOA, we specifically measured the arrival of ricin to the TGN by the sulfation assay. This assay takes advantage of the *trans*-Golgi-specific sulfation process, in which tyrosyl protein sulfotransferases couple sulfate groups to proteins displaying a sulfation site [26]. By addition of recombinant ricin molecules containing a sulfation site, the arrival of toxin molecules to the Golgi can be detected. Indeed, treatment with increasing concentrations of OHOA potentiated the ricin sulfation (Figure 4C). The total amount of ricin associated with the cells was unchanged. To address the specificity of the stimulatory effect of OHOA, unmodified OA was used in parallel. In agreement with the toxicity data, OA had a very limited effect on ricin sulfation (Figure 4C).

To detect the arrival of ricin to the Golgi by an alternative approach, we took advantage of a recently established method based on a Golgi-localized SNAP-tag coupling assay [27]. The SNAP-tag enzyme is maintained in the *trans*-Golgi through fusion to the C-terminal part of galactosyl transferase, and benzylguanine-labeled cargo molecules that are transported to the Golgi are covalently coupled to the SNAP-tag (Supplementary Figure S6). We thus labeled ricin with benzylguanine (BG) and detected the coupling of ricin to the Golgi-localized SNAP-tag by immunoblotting. In line with the sulfation data, treatment with OHOA increased the amount of ricin that was captured by the SNAP-tag in a concentration-dependent manner (Figure 4D). Furthermore, to establish a transport assay even more comparable to the sulfation assay, we fused the C-terminal part of tyrosyl protein sulfotransferase 1 [28] to the SNAP-tag enzyme (Supplementary Figure S7A, S7B). A comparable concentration-dependent increase in ricin capture upon treatment with OHOA was detected also by this procedure (Supplementary Figure S7C). Importantly, treatment with OHOA did not alter the structure of the Golgi complex itself, as visualized by Golgi-specific markers (Supplementary Figure S8).

Along the same lines, and as previously shown for cholera toxin [29], we established a SNAP-tag assay for measuring the arrival of cargo molecules to the ER. The SNAP-tag enzyme was maintained selectively in the ER lumen by fusion to an ER-signal peptide and the KDEL-retrieval sequence (Supplementary Figure S9). In line with the data for Golgi-transport, ricin transport to the ER was stimulated by OHOA in a concentration-dependent manner (Figure 4E).

To see whether the OHOA-induced stimulation of retrograde transport was specific to ricin, we measured the retrieval of the cation-independent mannose-6-phosphate receptor (CIMPR) from the cell surface to the Golgi by using a HeLa cell line expressing CIMPR fused to CD8 as previously described [30]. In line with the ricin data, the retrieval of CIMPR was slightly stimulated by treatment with OHOA (Supplementary Figure S10). Furthermore, as OHOA is known to induce changes in membrane microdomain organization [9], and lipid raft integrity is important for proper retrograde transport of glycosphingolipid-binding cargo, such as Shiga toxin (Stx) [31] and cholera toxin [32], we wanted to know whether treatment with OHOA would alter the retrograde transport of Stx. Stx transport to the Golgi was not stimulated by OHOA, but rather reduced (Supplementary Figure S11). Such a differential effect on ricin- and Stx transport is in line with data obtained from HEp-2 cells upon treatment with polyunsaturated fatty acids [33] or upon depletion of sphingolipids [34]. Thus, the retrograde transport of glycosphingolipids and bulk membrane are differentially sensitive to membrane lipid alterations [16].

### Incorporation of OA and OHOA into phospholipids, diacylglycerol and triacylglycerols

**Figure 5:**
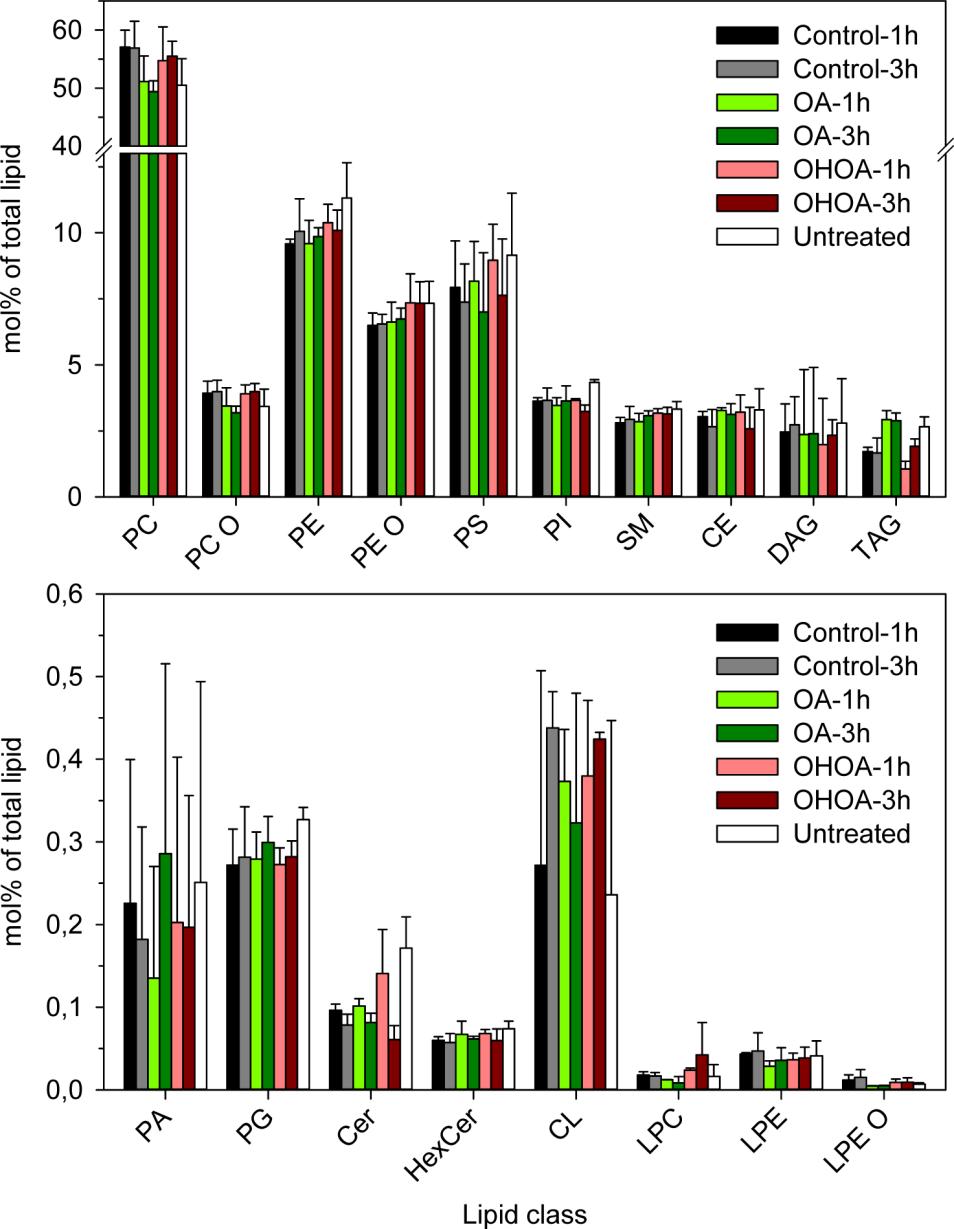
Lipidomics of HeLa cells treated with OHOA or OA HeLa cells were treated with 12.5 μM OHOA or OA for 1 or 3 hours before the cells were subjected to lipidomic analysis as detailed in Materials and Methods. The bars show the mol% of each lipid class (see Materials and Methods for abbreviations) as percentage of the total amount of lipids quantified; mean values + SEM quantified from 3 independent experiments.

The above data revealed that OHOA had a more pronounced stimulatory effect on ricin transport than the unmodified OA. We wondered if analyses of the cellular lipidome would reveal changes that could help in explaining these differences, and we therefore performed lipidomic analyses of HeLa cells treated with low concentrations of OA or OHOA for 1 or 3 hours. As shown in Figure 5, only minor changes were observed for most lipid classes following incubation with OA or OHOA. The major differences were observed for cells incubated with OA, where a large increase of triacylglycerol (TAG), a substantial increase of DAG, and a decrease of phosphatidylcholine (PC) were obtained. Most of the increase in TAG was due to an increase in TAG 54:3, i.e. most likely TAG with 3 OA (3 × 18:1), although some increase was also due to TAG 52:2, i.e. most likely due to TAG with C16:0 and 2 OA (data not shown). The increase in DAG with OA was due to an increase in DAG 18:1/18:1 (data not shown).

The changes in the lipidome were much less pronounced in cells incubated with OHOA; the small increase observed for TAG was mainly due to an increase of TAG 52:2:1, i.e. most likely TAG containing one each of the fatty acyl groups of 16:0, 18:1 and OHOA (for nomenclature, see Materials and Methods). We have earlier reported that changes in DAG may be of importance for intracellular transport [35]. We observed a decrease in DAG after 1 hour of incubation with OHOA (down to 62% of control; *p* = 0.013), whereas the DAG level increased to 115% of the control (*p* = 0.66) after 3 hours of incubation with OHOA. It seems unlikely that these changes in DAG-levels could explain the OHOA-stimulated transport of ricin.

We estimated the amount of OHOA incorporated into phospholipids and TAG to constitute close to 6% of the total lipidome after 3 hours of incubation (Table 1), with some increase over time (data not shown). These data are in the same range as reported by Martin *et al*. [10] for incorporation of OHOA into three other cell lines. Notably, we did not detect any changes in total SM or in the relative contribution of SM species upon short-term treatment with OHOA (Supplementary Figure S12). Moreover, no changes were detected in the levels of either free or total cholesterol (including cholesteryl esters) upon treatment with OHOA or OA (Supplementary Figure S13). This is in accordance with data from U118 cells [15].

**Table 1:** Incorporation of OHOA into lipid classes

Lipid classes	Incorporation of OHOA into lipid classes after 3 h incubation (Mean and range; % of total)
PC	8.2% (7.5–8.7)
PC O	9.0% (7.4–10.2)
PE	3.1% (2.7–3.6)
PE O	0.6% (0.4–0.7)
TAG	28% (10–59%) ^a^
Sum^b^	∼5.8%

a There is large variation in these data as the amount of TAG containing OHOA (TAG 52:2;1, i.e. most likely TAG containing one each of the fatty acyl groups of 16:0, 18:1 and OHOA) was less than 10 pmol.

b The mol% of the above lipid classes in OHOA-treated cells (see Figure 5) was used to calculate this sum: 55% PC, 4% PC O, 10% PE, 7% PE O and 1% TAG.

### OHOA-stimulated ricin transport requires cellular signaling and amphitropic proteins

**Figure 6:**
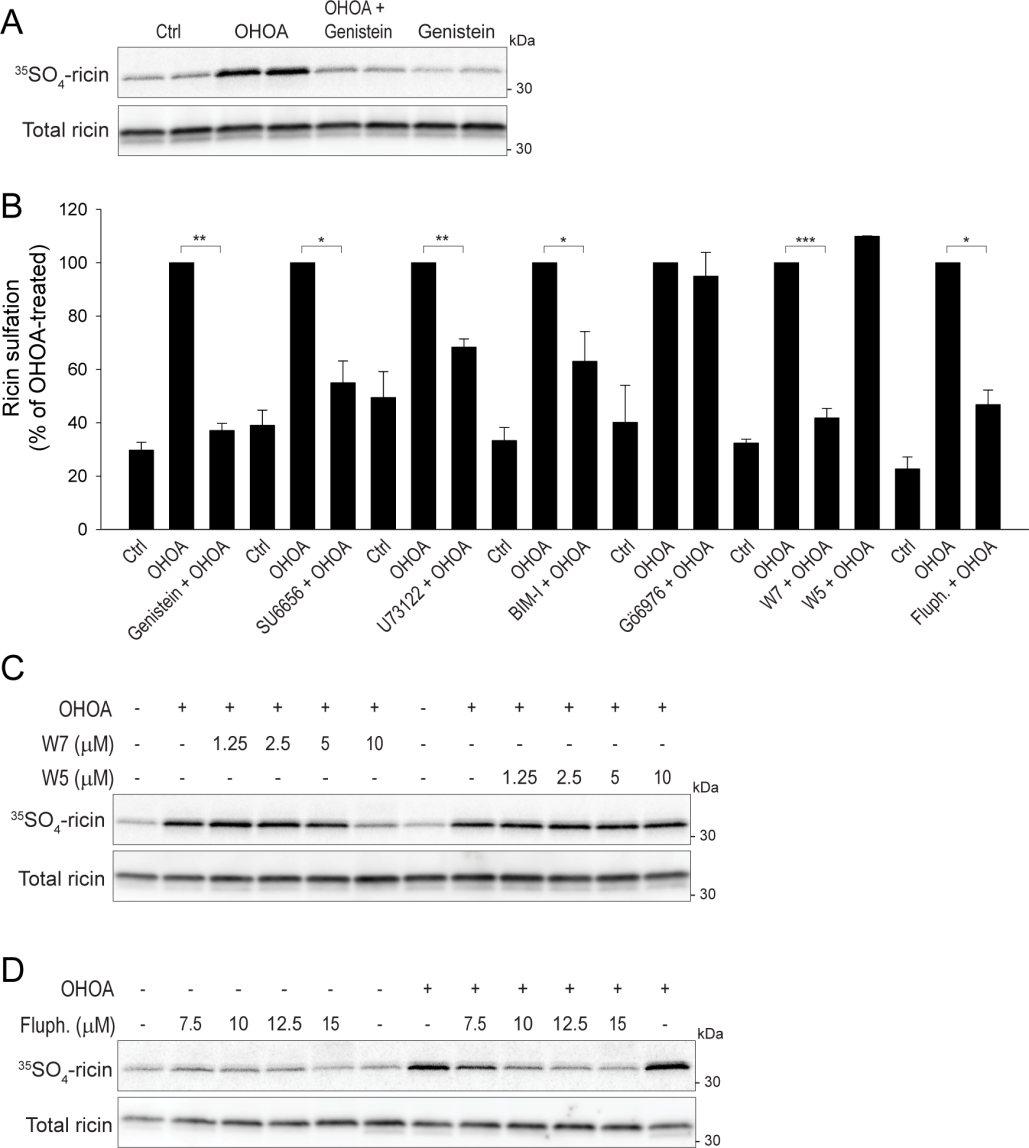
OHOA-stimulated ricin transport requires cellular signaling (**A**) HeLa cells were incubated with 12.5 μM OHOA in the absence or presence of 25 μg/ml genistein and assayed for ricin sulfation as described in Materials and Methods. A representative sulfation autoradiograph with the corresponding immunoblot is shown. (**B**) HeLa cells were incubated with 12.5 μM OHOA in the absence or presence of 5 μM SU6656, 10 μM U73122, 10 μM BIM-I, 5 μM Gö6976, 10 μM W7, 10 μM W5, or 10 μM fluphenazine (fluph) and assayed for ricin sulfation. The band intensities were quantified and normalized to the OHOA-alone-value. The bars show mean values ± SEM quantified from at least 3 independent experiments; *p <0.05; **p < 0.01; ***p < 0.001. (**C**–**D**). HeLa cells were incubated with 12.5 μM OHOA and the indicated concentrations of W7 and W5 (C), or fluphenazine (D), and assayed for ricin sulfation. Representative sulfation autoradiographs with the corresponding immunoblots are shown.

Having established that OHOA both activates several key signaling pathways and stimulates ricin transport, we wanted to know whether these two processes are related. First, to broadly block cellular signaling we used the general tyrosine kinase inhibitor genistein. Indeed, genistein treatment strongly inhibited the OHOA-stimulated ricin sulfation (Figure 6A, 6B), and a similar inhibition was detected by the Golgi SNAP-tag assay (Supplementary Figure S14A).

To more specifically address which signaling pathway(s) are involved in OHOA-induced ricin transport, we performed the ricin sulfation assay in the presence of a range of kinase inhibitors. First, we wanted to see whether the OHOA-induced activation of AKT1 or MTOR promotes ricin transport. Although the AKT1 inhibitor VIII completely inhibited the basal AKT1 phosphorylation at all concentrations used, the OHOA-induced ricin sulfation was unaltered (Supplementary Figure S14B). Likewise, the MTOR inhibitor Torin1 did not reduce the OHOA-stimulated ricin sulfation (Supplementary Figure S14C). Next, we assessed whether MAPK14 or MAPK8 are important for ricin sulfation. When the MAPK14 inhibitor SB203580 or the MAPK8 inhibitor JNK-IN-8 were used at concentrations where the kinases were fully inhibited (Supplementary Figure S14D, S14F), the OHOA-stimulated ricin sulfation was virtually unchanged (Supplementary Figure S14E, S14G, S14H). Likewise, the EGFR/ERBB2 inhibitor lapatinib did not reduce OHOA-stimulated ricin sulfation (Supplementary Figure S14I), which is in line with the lack of EGFR-family activation by OHOA.

As OHOA has been suggested to affect membrane recruitment of amphitrophic proteins, we wanted to see whether key signaling proteins regulated by amphitropism are involved in ricin transport. First, we tested the Src family kinase inhibitor SU6656, and indeed, the OHOA-stimulated ricin transport was strongly reduced (Figure 6B; Supplementary Figure S14J). Similarly, the PLC inhibitor U73122 reduced OHOA-stimulated ricin sulfation (Figure 6B; Supplementary Figure S14K). This might be explained by a PLC-induced release of Ca^2+^ from intracellular stores, as local Ca^2+^ fluxes have been implicated in intra-Golgi transport [36] and Ca^2+^ is known to regulate ricin retrograde transport [37]. Moreover, both DAG and Ca^2+^are important activators of PKC, and as OHOA was shown to promote translocation and activation of PKCα in A549 cells [4], we tested if PKC activity would be important for ricin transport. OHOA-induced ricin sulfation was partially reduced by the general PKC inhibitor BIM-I (Figure 6B; Supplementary Figure S14L), but unaltered by the PKC α/β inhibitor Gö6976 (Figure 6B). Thus, although PKC activity might be partially involved in OHOA-induced ricin transport, a role for PKCα seems less likely.

Recruitment of amphitropic proteins not only stimulates signaling, but can also promote membrane transformations, such as tubule formation or vesicle scission. One potential candidate is dynamin, which might be partially regulated by amphitropic mechanisms due to membrane insertion of its PH domain [38], and is known to regulate the retrograde transport of both ricin [18] and Stx [39]. Inhibition of dynamin by Dyngo-4a led to a total block not only of basal ricin sulfation as expected [18], but also of OHOA-induced ricin sulfation (Supplementary Figure S15), suggesting that also the OHOA-induced ricin sulfation is dynamin-dependent. Other relevant proteins regulated by amphitropism are members of the SNX family, which contain a BAR (Bin, Amphiphysin, RVS) domain, and are able to induce, sense and/or stabilize membrane curvature, as reviewed in [26]. As SNXs are implicated in ricin transport from endosomes to the Golgi [17], we wanted to see whether OHOA would alter the recruitment of SNXs to early endosomes. To this end we quantified the amount of SNX1 or SNX2 localized to EEA1-positive endosomes upon treatment with OHOA, but found no significant changes in endosomal localization (Supplementary Figure S16). As SNXs can take part in the retromer complex involved in transport between endosomes and the Golgi [26], we also stained for the retromer components VPS26 and VPS35. Interestingly, OHOA increased the endosomal localization of VPS35, and a similar trend was observed for VPS26, although the data did not reach statistical significance (Supplementary Figure S17). Notably, treatment with OHOA did not affect the size of the EEA1-positive endosomes themselves (data not shown), and we could not detect any increased tubulation of the endosomes or other morphological changes.

The translocation of amphitropic proteins to and from membranes is not only regulated by membrane properties, but also by binding of ligands, such as Ca^2+^ or nucleotides, and protein-interactions, for example to calmodulin [6]. Calmodulin is activated by Ca^2+^ and is known to regulate various membrane fusion events [40]. As Ca^2+^ promotes Golgi transport of ricin [37], we wanted to elucidate the role of calmodulin in this transport step. The calmodulin inhibitor W7 reduced the OHOA-stimulated ricin sulfation in a concentration-dependent manner (Figure 6B, 6C), whereas W5, which is structurally related to W7 but significantly less potent at inhibiting calmodulin activity, had no effect. A similar inhibition was obtained with the calmodulin inhibitor fluphenazine (Figure 6B, 6D). Importantly, both W7 and fluphenazine seemed to selectively target the OHOA-stimulated ricin transport and did not reduce the unstimulated, basal ricin transport in these cells (Figure 6D and data not shown). These data might indicate that calmodulin selectively regulates the OHOA-stimulated retrograde transport of ricin.

## DISCUSSION

In the present study we demonstrate that the membrane lipid therapy agent OHOA rapidly induces cellular signaling and stimulates retrograde membrane transport in a process that seems to involve amphitropic proteins and Ca^2+^/calmodulin.

Our data show that OHOA within the first few minutes after addition to cells stimulates release of intracellular Ca^2+^ and activates PI3K. Although these are novel effects of OHOA, the findings are in line with data obtained in cells treated with unmodified OA. In breast cancer cells, OA was shown to stimulate proliferation [20, 41], and to rapidly increase cytosolic Ca^2+^ and activate PI3K and AKT1 [41]. PI3K and AKT1 were also activated by OA in the hepatoma cell line Huh-7 [42]. Both in the above studies and in our experiments, the fatty acids OA and OHOA appear to act as growth factors. This might be explained by the fact that the presence of fatty acids can be regarded as an indicator of nutritional uptake.

Fatty acids are believed to interact with and have the ability to signal through a variety of cell surface receptors, and one potential target is the EGFR. Proliferation of MCF-7 breast cancer cells was activated by OA, most likely via transactivation of the EGFR [20], and OA was shown to downregulate ERBB2 in breast cancer cells [21]. Moreover, several unsaturated fatty acids, including OA, have been shown to stimulate the EGFR in a ligand-independent manner [19]. Interestingly, even mild surfactants were shown to activate the EGFR, and the receptor was thus suggested to act as a sensor for amphiphiles and membrane fluidity changes [19]. Our data do not support a role for the EGFR in the OHOA-induced signaling in HeLa cells, as we did not detect any activation or internalization of the EGFR by treatment with OHOA, and moreover, OHOA-stimulated signaling was unaltered by the dual EGFR/ERBB2 inhibitor lapatinib. Although we cannot exclude whether OHOA would have a long-term effect on the expression level of EGFR family members in HeLa cells, the acute drug-induced signaling does not seem to be mediated via the EGFR family.

Alternatively, OHOA has the potential to interact with two G-protein coupled receptors that have recently been identified as receptors for medium- and long-chain fatty acids, namely FFAR1/Gpr40 and FFAR4/Gpr120 (reviewed in [43]). Interestingly, it was found that the OA-stimulated Ca^2+^ flux and activation of PI3K and AKT1 in MDA-MB-231 cells was not downstream of the EGFR, but rather mediated in part by recognition of OA by FFAR1 [44]. In a similar manner, OA was shown to induce proliferation and MAPK1/3 activity in MCF-7 cells via transactivation of the EGFR, possibly after initial stimulation of the FFAR1/4 [20]. Moreover, OA was found to stimulate PI3K and AKT1 via FFAR4 in Huh-7 cells [42]. It remains to be seen whether the OHOA-induced signaling might be mediated via members of this class of receptors.

In contrast to the stimulatory effects of OHOA demonstrated in this paper, treatment with OHOA has previously been shown to downregulate pro-survival cellular signaling in glioma cells by translocation of Ras from the plasma membrane to the cytosol [14]. These differential results might be explained by cell type differences, as the cellular lipid composition and the membrane microdomain organization have been suggested as determining factors for the mechanism of action of OHOA in each particular cell type [1]. OHOA was shown to be particularly efficient in glioma cells, which were found to have a subnormal expression level of SM [14, 15]. Treatment with OHOA activated SM synthase, which led to restoration of SM to normal levels and induction of cell death [15]. In contrast, during the short-term treatments in the present study (up to 3 hours) both the total level of SM and the level of different SM species remained unaltered. Thus, SM synthase activation does not seem to contribute to our observed phenotypes in HeLa cells.

How might OHOA-mediated alterations of membrane properties explain the increased signaling and retrograde transport observed in the present study? It is known that membrane incorporation of *cis*-unsaturated fatty acids with kinked acyl chains induces lipid packing defects and increased water penetration into the bilayer [5]. Such factors would weaken the lipid-lipid interactions, and thus promote intercalation of amphipathic helices of peripheral membrane proteins into the bilayer. As an example, membrane binding of the amphitropic protein CTP:phosphocholine cytidylyltransferase is promoted by factors that increase the phospholipid head group spacing, such as high curvature, the presence of fatty acids or DAG, which both have small “head groups”, and the presence of phospholipids with oxidized acyl chains [45]. As our lipidomics-data revealed that OA was incorporated into complex lipids to a much higher extent than OHOA, it seems unlikely that the *cis*-unsaturated fatty acid tail of OHOA is solely responsible for our observed phenotypes. Thus, we speculate that the hydroxyl-group of OHOA might contribute to our cellular effects by alterations in membrane properties via increased charge or altered head group area. In addition, incorporation into phospholipids removes the charge of a fatty acid, thus if free OHOA remains longer in the membrane than free OA due to reduced metabolism, such membranes could potentially have a more anionic surface, which could contribute further to altered recruitment of amphitropic proteins.

OHOA-stimulated ricin transport seemed to partially depend on kinases potentially regulated by amphitropism. Amphitropic proteins might regulate membrane transport by either promoting signaling, or membrane transformations. Interestingly, Src has previously been implicated in Golgi trafficking via a cargo-activated signaling cascade initiated on the Golgi complex itself [46]. Binding of cargo to the KDEL-receptor was found to activate Src via the G-protein Gα_q/11_ [47], and the authors speculated that this mechanism of activation could include a G-protein-stimulated local release of Ca^2+^ from intracellular stores. Along these lines, it is tempting to speculate that if OHOA has the ability to activate Golgi-localized G-proteins, OHOA-activated Ca^2+^ release and Src activity might promote Golgi transport.

Alternatively, altered recruitment of membrane transforming proteins might lead to increased generation of transport vesicles or tubules, or promote fusion and fission processes. For example epsinR is involved in retrograde transport from the endosomes to the Golgi, and insertion of the amphipathic helix of epsin has been shown to deform membranes and promote fission in a dynamin-independent manner [48]. Also proteins involved in membrane scission, such as dynamin and EHD proteins, possess hydrophobic patches that might contribute to membrane recruitment, and both dynamin [18, 39] and EHD proteins [49, 50] are shown to regulate retrograde transport. Moreover, the hydrophobic ALPS motif, which is the most sensitive membrane curvature sensor, recognizes lipid packing defects arising from membrane bending, i.e. positive curvature [51, 52]. Interestingly, binding of ALPS to model membranes could be mimicked by introducing *cis*-unsaturated, conical lipids into a flat lipid bilayer [53]. Based on this, it is tempting to speculate that OHOA in a similar manner is able to induce lipid packing defects that could mimic positively curved membranes, which might promote recruitment of proteins with curvature-sensing recognition motifs, such as the BAR domain-containing SNXs [26]. Although OHOA did not promote endosomal recruitment of SNX1 or SNX2, we detected increased endosomal localization of the retromer component VPS35. Thus, the possibility exists that OHOA promotes ricin transport by increasing the membrane affinity for proteins mediating retrograde trafficking.

A subset of BAR domain proteins, termed N-BAR proteins, are able to sense lipid packing defects via an amphipathic helix N-terminal to the BAR domain. Interestingly, several N-BAR domains, including those of amphiphysin and endophilin, were recently shown to bind calmodulin, which was found to promote tubulation and regulate the activity of the N-BAR protein [54]. Whether calmodulin promotes tubulation of endosomes for sorting into the retrograde pathway via interactions with N-BAR proteins remains to be seen, but such a scenario could contribute to the OHOA-induced ricin transport observed in the present study. As calmodulin has been shown to regulate endosome fusion [36] and intra-Golgi transport [55], and to be implicated in SNARE-mediated fusion [56], the OHOA-stimulated ricin transport might also be explained by increased fusion at the endosome level or increased fusion between incoming vesicles and the Golgi complex.

In conclusion, treatment of cells with OHOA stimulates several signaling pathways and activates retrograde membrane transport in a process dependent on calmodulin and several proteins regulated by amphitropism. This study shows that addition of a lipid known to alter membrane properties not only affects signaling, but also intracellular transport.

## MATERIALS AND METHODS

### Materials

EGF, genistein, genistin, fluphenazine, concanamycin A (ConA), bisindolylmaleimide (BIM) I, U73122, brefeldin A (BFA), and thapsigargin were from Sigma-Aldrich. SU6656, W7, W5, and AKT1 inhibitor VIII were from Calbiochem. Lapatinib was from LC Laboratories, ^125^I-EGF from Perkin Elmer, bafilomycin A1 (BafA1) from Enzo Life Sciences, Torin1 from Tocris Bioscience, Dyngo-4a was from Abcam Biochemicals, and H_2_^35^SO_4_ was purchased from Hartmann Analytics. All other chemicals were from Sigma-Aldrich or Merck unless otherwise stated. The environment-sensitive probe NR12S was a kind gift from Prof. A. Klymchenko (University of Strasbourg, Strasbourg, France).

### Cells

The cell lines HeLa, HEp-2 and U2OS were obtained from ATCC, they were all authenticated, and were grown under 5% CO_2_ in DMEM supplemented with 10% fetal calf serum, 100 U/ml penicillin, 100 μg/ml streptomycin and 2 mM L-glutamine (all from Gibco). For treatment with fatty acids, the cells were washed twice in Hepes-buffered MEM and starved for 90 minutes in Hepes-buffered MEM containing 3 μM fatty-acid free BSA before addition of fatty acids. The acid form of OHOA was a kind gift from Pablo Escribá (Lipopharma Therapeutics), and was dissolved in DMSO to a stock concentration of 100 mM. The sodium salt of OHOA (Avanti Polar Lipids) and OA (Sigma-Aldrich) were dissolved in 50% ethanol to a 50 mM stock solution. Identical data were obtained with either the acid form or the sodium salt of OHOA (data not shown), however, when OHOA and OA were used in parallel, the sodium salt of both drugs were used. Generally for inhibitor studies, cells were pretreated with the inhibitor at the indicated concentration for 30 minutes before addition of OHOA.

### Spectral GP imaging on confocal microscope

HeLa cells were seeded in 8-well Lab-Tek chambered coverglass at a density of 1.8 × 10^4^ cells/well one day before the experiment. The cells were washed twice with warm Hepes-buffered Live Cell Imaging Solution (#A14291DJ, ThermoFisher Scientific) supplemented with 20 mM glucose and then incubated with 50 μM OHOA or 0.05% ethanol or with Live Cell Imaging Solution alone for 30 minutes at 37°C. As a positive control for an increase in membrane fluidity, cells were treated with 5 mM methyl-β-cyclodextrin (mβCD) for 1 hour at 37°C, and then medium was replaced with fresh Live Cell Imaging Solution. To stain cell membranes with NR12S probe, NR12S was diluted in Live Cell Imaging Solution and immediately added to cells resulting in the final dye concentration of 10 nM. After 7 minutes, the imaging was started and continued for 10 minutes resulting in 7–8 images taken per condition. The images were acquired using Zeiss LSM 780 confocal microscope equipped with a 63x objective, NA 1.4 and a 32-channeled GaAsP detector array. Laser light at 514 nm was used for the excitation of NR12S, and the fluorescence detection range was set between 521 and 687 nm with 8.7 nm intervals. The focus was set to equatorial plane of the cells slightly above the cover glass surface. The sample chamber was controlled for temperature and kept at 37°C. Spectra for each image pixel were obtained from the intensity values of the different detection channels by using the ImageJ software function “Plot Z-axis profile” [57]. The analysis was performed on manually selected regions of the plasma membrane which were not in contact with other cells (to avoid any effects of cell-to-cell contacts). The background signal was determined by applying selections of the same size on a dark region of the image, which was subsequently subtracted from the values obtained from the regions of interest. At least 20 regions of interest were analyzed per condition for each independent experiment. The average GP value for each selected region was calculated according to equation: $\text{GP}=\frac{I(573)-I(599)}{I(573)+I(599)}$, where I(573) and I(599) are the fluorescence intensities at wavelengths 573 nm and 599 nm, respectively. The intensity values directly obtained from spectra are arbitrary, and thus the GP values are consistent only within one experimental study, and cannot be directly compared to values obtained with other dyes or imaging settings. Therefore, a difference in GP value between the treatment and the control rather than the absolute GP value was used for the final quantification: ΔGP *=* GP(treatment) − GP(control).

### Calcium release assay

Changes in intracellular calcium were measured using the Fluo-4 NW calcium Assay Kit (Molecular Probes) according to the manufacturer's recommendations. In brief, HeLa cells were seeded in 96-well plates at a density of 1 × 10^4^ cells/well one day before the experiment. The cells were then starved in Hepes-buffered medium for 1.5 hours, before being loaded with cell-permeable Fluo-4 dye for 40 minutes. OHOA or OA (or EtOH as control) was then automatically added using a syringe dispenser, to a final concentration of 25 μM. When indicated, the assay was performed in a calcium-free Hanks’ balanced salt solution (Gibco) supplemented with 20 mM Hepes and 0.5 mM MgCl_2_. After addition of the desired compounds, the fluorescence was measured every 20 seconds, 2 minutes before and 10 minutes after addition, using a Synergy2 plate reader (BioTek) with 485/20 and 528/20 excitation and emission filters, respectively. Four replicate wells were measured at each time point, and the results are presented as change in fluorescence over time relative to pre-injection fluorescence.

### Immunoblotting

Treated cells were washed with PBS and lysed (0.1 M NaCl, 10 mM Na_2_HPO_4_, pH 7.4, 1 mM EDTA, 1% Triton X-100, 60 mM n-octyl β-D-glucopyranoside, and cOmplete EDTA-free Protease Inhibitor Cocktail (Roche)). The cleared lysates were separated by 4–20% SDS-PAGE and transferred to a PVDF membrane. The membrane was blocked in 7.5% milk for 45 minutes, followed by overnight incubation with the indicated antibodies in 5% BSA, 45 minutes incubation with HRP-conjugated secondary antibodies and detection with SuperSignal West Dura Extended Duration Substrate (Thermo Scientific) in a ChemiDoc Imaging System (Bio-Rad). The signal intensities were quantified by the Quantity One software (Bio-Rad) and were normalized to the loading control. The following antibodies were used: phospho-AKT1^Ser473^ (#9271), AKT1 (#4691), phospho-RPS6KB^Thr389^ (#9205), RPS6KB (#9202), phospho-Erk/ MAPK1/3^Thr202/Tyr204^ (#9106), Erk/MAPK1/3 (#9102), phospho-p38/MAPK14^Thr180/Tyr182^ (#9211), phospho-JUN (#9164), ERBB2^Tyr877^ (#2241), PERK /EIF2AK3 (#5683) all from Cell Signaling Technology, and p-38/MAPK14 (#612168, Transduction Laboratories), GFP (clone 3H9, ChromoTek), phospho-EGFR^Tyr1173^ (#ab24912, Abcam), GAPDH (#ab9482, Abcam), ACTIN (#CLT9001, Cedarlane), SNAP (#P9310S, New England Biolabs), Shiga toxin (#STX1-3C10, Toxin Technology), ricin (#C86370M, Meridian), and XBP1s (#647502, BioLegend).

### Lipid analyses

HeLa cells were washed in Hepes-buffered MEM medium and starved for 90 minutes in Hepes-buffered MEM before addition of 12.5 μM OA, OHOA or vehicle (50% EtOH). The incubation was continued for 1 or 3 hours at 37°C, then the cells were detached by trypsin/EDTA, washed in PBS and counted. 4.5 × 10^5^ cells per sample were sent for lipid analyses. The experiment was independently performed three times with duplicates. The sample named “Untreated” was neither starved, nor treated.

Mass spectrometry-based lipid analysis was performed as described [58]. Lipids were extracted using a two-step chloroform/methanol procedure as in Ejsing *et al*. [59]. Samples were spiked with internal standard lipid mixture containing: with internal standard lipid mixture containing: cardiolipin 16:1/15:0/15:0/15:0 (CL), ceramide 18:1;2/17:0 (Cer), cholesterol D6 (chol), diacylglycerol 17:0/17:0 (DAG), hexosylceramide 18:1;2/12:0 (HexCer), lyso-phosphatidylcholine 12:0 (LPC), lyso-phosphatidylethanolamine 17:1 (LPE), lyso-phosphatidylglycerol 17:1 (LPG), lyso-phosphatidylinositol 17:1 (LPI), lyso-phosphatidylserine 17:1 (LPS), phosphatidate 17:0/17:0 (PA), phosphatidylcholine 17:0/17:0 (PC), phosphatidylethanolamine 17:0/17:0 (PE), phosphatidylglycerol 17:0/17:0 (PG), phosphatidylinositol 16:0/16:0 (PI), phosphatidylserine 17:0/17:0 (PS), cholesterol ester 20:0 (CE), sphingomyelin 18:1;2/12:0;0 (SM) and triacylglycerol 17:0/17:0/17:0 (TAG). After extraction, the organic phase was transferred to an infusion plate and dried in a speed vacuum concentrator. 1st step dry extract was re-suspended in 7.5 mM ammonium acetate in chloroform/methanol/propanol (1:2:4, V:V:V) and 2nd step dry extract in 33% ethanol solution of methylamine in chloroform/methanol (0.003:5:1; V:V:V). All liquid handling steps were performed using Hamilton Robotics STARlet robotic platform with the Anti Droplet Control feature for organic solvents pipetting. Samples were analyzed by direct infusion in a QExactive mass spectrometer (Thermo Scientific) equipped with a TriVersa NanoMate ion source (Advion Biosciences). Samples were analyzed in both positive and negative ion modes with a resolution of R_m/z=200_ = 280000 for MS and R_m/z=200_ = 17500 for MSMS experiments, in a single acquisition. MSMS was triggered by an inclusion list encompassing corresponding MS mass ranges scanned in 1 Da increments. Both MS and MSMS data were combined to monitor CE, DAG and TAG ions as ammonium adducts; PC, PC O-, as acetate adducts; and PA, PE, PE O-, PG, PI and PS as deprotonated anions. MS only was used to monitor LPE, LPE O-, LPI and LPS as deprotonated anions; Cer, HexCer, SM, LPC and LPC O- as acetate adducts and cholesterol as ammonium adduct of an acetylated derivative. The additional “O”, i.e. for PC O-, PE O- and LPE O- means that these lipids contain an ether-linked chain, either an alkyl or alkenyl. The presence of OHOA was confirmed by MSMS. Data were analyzed with in-house developed lipid identification software based on LipidXplorer [60, 61]. Data post-processing and normalization were performed using an in-house developed data management system. Only lipid identifications with a signal-to-noise ratio >5, and a signal intensity 5-fold higher than in corresponding blank samples were considered for further data analysis.

When discussing different lipid species the following annotations are used: Lipid class-<sum of carbon atoms>:<sum of double bonds>; < sum of hydroxyl groups>, i.e. SM-34:1;2 means an SM species with 34 carbon atoms, 1 double bond and 2 hydroxyl groups in the ceramide backbone. This SM species is most likely SM d18:1/16:0, but we cannot exclude other possibilities as fragmentation data were not obtained.

### Cholesterol assay

Cells grown overnight were starved for 1.5 hours in Hepes-buffered medium before incubation with 12.5 μM OHOA or OA (or EtOH as control) for 1 or 3 hours at 37°C. The cells were then washed in pre-warmed PBS, followed by 5 minutes incubation in lysis buffer (0.1% SDS, 0.1 M TRIS, 0.001 M EDTA, pH 7.4) at 37°C. The cell lysate was homogenized by repeated pipetting through a 19 G needle, before being processed for cholesterol measurements using an Amplex Red Cholesterol Assay Kit (Molecular Probes) according to the manufacturer's recommendations. In brief, the cell lysate was diluted 2× in reaction buffer and one pair of duplicates from each sample was incubated with Amplex red reagent containing HRP and cholesterol oxidase to detect free cholesterol, whereas another pair of duplicates was incubated with cholesterol esterase in addition to measure the total cholesterol content of the samples, i.e. both free cholesterol and cholesteryl esters. The fluorescence was measured using a Synergy2 plate reader (BioTek, VT, USA) with 530/25 and 590/35 excitation and emission filters, respectively. The cholesterol concentration of each sample was calculated based on a cholesterol standard curve generated in the same experiment, and normalized to the total protein content as measured by the BCA assay (Thermo Scientific). 2 replicate wells were measured per condition, and the results are presented as fold change relative to the control. The experiment was performed twice.

### Ricin toxicity, endocytosis, recycling and degradation

The toxicity of ricin was measured as protein synthesis inhibition as previously described [34]. Endocytosis, recycling or degradation of ^125^I-ricin was measured as previously described [34]. The cells were pretreated with OA or OHOA for 30 minutes before addition of ricin.

### Sulfation of ricin and shiga toxin

The sulfation experiments were performed essentially as previously described [25]. Briefly, cells were preincubated in sulfate-free DMEM containing 0.2 mCi/ml H_2_^35^SO_4_ for 2 hours at 37°C with inhibitors present for the last hour and OA/OHOA present for the last 30 minutes. Then ricin-sulf1 or ShigaB-sulf2 was added and the incubation continued for 1 hour (Stx) or 1.5 hours (ricin) at 37°C. Surface-bound ricin was removed by 0.1 M lactose, then the cells were washed in cold PBS, lysed (0.1 M NaCl, 10 mM Na_2_HPO_4_, pH 7.4, 1 mM EDTA, 1% Triton X-100, 60 mM n-octyl β-D-glucopyranoside, and cOmplete EDTA-free Protease Inhibitor Cocktail (Roche)) and scraped. The cleared lysate was subjected to immunoprecipitation with antibodies against Stx or ricin prebound to protein-A/Sepharose CL-4B (GE Healthcare) overnight at 4°C. The adsorbed material was analyzed by 4–20% SDS-PAGE under reducing conditions and transferred onto a PVDF membrane. The bands were detected by exposing the membrane to a K-Screen (Bio-Rad), and the signal intensities were quantified by the Quantity One software (Bio-Rad). The total amount of cell-associated toxin was determined by immunoblotting of the same membrane.

### Generation of stable cell lines

Stable HeLa cell lines were generated by lentiviral transduction. The GalT-GFP-SNAP cell line was based on a plasmid encoding the first 120 amino acids of galactosyl transferase (GalT) fused to EGFP and SNAP, which was a kind gift from Prof. Ludger Johannes (Pasteur Institute, France). To generate the TPST1-GFP-SNAP cell line a sequence encoding the first 37 amino acids of tyrosyl protein sulfotransferase 1 (TPST1) was fused to GFP-SNAP. This sequence is sufficient for localizing the enzyme to the *trans*-Golgi [28], and the plasmid was generously provided by Prof. Peter Bayer (Universität Duisburg-Essen, Germany). The ER localized SNAP cell lines were based on an ER-mCherry-KDEL plasmid obtained from Prof. Harald Stenmark (Oslo University Hospital, Norway). The mCherry sequence was first substituted by EGFP-SNAP to produce an ER-EGFP-SNAP-KDEL cell line. Then also an ER-mCherry-SNAP-KDEL cell line was generated by substituting EGFP by mCherry. The various SNAP plasmids were subcloned into a Gateway ENTRY vector by standard molecular biology techniques. From this vector, lentiviral transfer vectors were generated by recombination with either pCDH-EF1a-GW-IRES-Bsd (a gateway-enabled derivative of pCDH-EF1a-MCS-IRES-Puro (Systems Biosciences) or pCDH-PGK-GW-IRES-Puro. Lentivirus particles were packaged using a third-generation packaging system (Addgene plasmid numbers 12251, 12253 and 12259) as previously described [62, 63]. Cells were then transduced with low virus titres (multiplicity of infection (m.o.i.) < 1) and stable cell pools were generated by selection with blasticidin (3 μg/ml) or puromycin (1 μg/ml). The localization of the SNAP-fusion protein was determined by immunofluorescence, and the SNAP enzymatic activity was confirmed by incubation with the fluorescent benzylguanine (BG), SNAP-Cell^®^ TMR-Star (#S9105S, New England Biolabs) (Supplementary Figures S6, S7, S9).

### SNAP-tagging the retrograde transport of ricin and Shiga toxin

Ricin and Shiga holotoxins were labeled with the SNAP-tag substrate BG-GLA-NHS (#S9151S, New England Biolabs) according to the manufacturer's instructions in molar ratios 1:1 and 3:1, respectively [27]. HeLa cells stably expressing the Golgi-localized GalT-GFP-SNAP or the TPST1-GFP-SNAP were incubated for 2 hours at 37°C in Hepes-buffered MEM medium in the presence or absence of drugs as for the sulfation assay, before 500 ng/ml BG-labeled toxins were added and the incubation continued for 1.5 hours at 37°C. The SNAP-tag reaction was stopped by incubation with SNAP-Cell^®^ Block at 1.25 mM (#S9106S, New England Biolabs) for 15 minutes at 37°C. This concentration was found to completely block the SNAP-tag reaction (data not shown). The following steps, cell lysis, immunoprecipitaion and SDS-PAGE, were performed exactly as described for the sulfation assay, or alternatively, the immunoprecipitation of Stx-SNAP complex was performed with antibodies against GFP. Toxin molecules coupled to the SNAP-tag were detected by immunoblotting, and the major band detected corresponds to one toxin subunit (A- or B-chain) coupled to the SNAP-fusion protein (Supplementary Figures S6, S7, S9). However, as previously observed [29], also weak bands of higher molecular weight complexes were detected, most likely corresponding to multimers.

For detection of ricin transport to the ER by the SNAP-tag method, BG-labeled ricin was labeled with ^125^I to increase the sensitivity of the method [64]. The SNAP-tag experiment and toxin immunoprecipitation was performed as described for the Golgi SNAP-tag assay above, except that ricin was present for 2 hours to allow time for ER-transport. The radioactive signals both from total- and SNAP-tagged ^125^I-ricin were detected by K-Screen (Bio-Rad), and the signal intensities were quantified by the Quantity One software (Bio-Rad).

### Immunofluorescence

Cells grown overnight on glass coverslips were starved for 1.5 hours in Hepes-buffered medium before incubation with OHOA as described in the figure legends. The cells were then washed with PBS, fixed in 10% formalin (Sigma Aldrich), washed again and permeabilized with 0.1% Triton X-100 for 2 minutes at room temperature. The cells were washed, followed by blocking in 5% FBS in PBS for 30 minutes. The cells were then incubated with the indicated antibodies for 1 hour at room temperature, followed by incubation with Alexa488-, Alexa568-, or Alexa647-labeled donkey IgGs (Jackson Immunoresearch Laboratories) for 1 hour at room temperature. After repeated washing, the cells were mounted with ProLong Gold antifade mounting medium with DAPI for nuclear staining (Molecular Probes). The cells were imaged using a Zeiss LSM780 laser scanning confocal microscope (Carl Zeiss MicroImaging) equipped with an Ar-Laser multiline (458/488/514 nm), a DPSS-561 10 (561 nm), and a Laser diode 405–30 CW (405 nm). The objective used was a Zeiss Plan-Apochromat 63×/1.40 Oil DIC M27. Images were acquired using the ZEN 2010 software (Carl Zeiss MicroImaging).

The following primary antibodies were used: EGFR (#20-ES04, Fitzgerald), EEA1 (#2411, Cell Signaling Technology, or #ab70521, AbCam), Giantin (#924302, Covance), TGN46 (#AHP500G, AbD Serotec), Calnexin (#MA3-027, Thermo Scientific), SNX1 (#611308, BD Biosciences), SNX2 (#611482, BD Biosciences), VPS26 (#ab23892, AbCam), and VPS35 (#ab10099, AbCam).

For determination of colocalization with EEA1, Fiji software was applied to quantify the fraction of each marker that localized to the area defined by the EEA1-staining (as percent of total cellular marker), and the data are presented as percent of untreated control. At least 40 cells were analyzed per condition per experiment.

### CIMPR retrograde transport

HeLa cells stably expressing the CD8-CIMPR fusion protein were a kind gift from Prof. Seaman, Cambridge, UK. To study retrograde transport of CIMPR these cells were stably transfected with GFP-tagged galactosyl transferase (GalT, aa 1–120). The protocol was slightly modified from Breusegem and Seaman [30]. Cells grown overnight on glass coverslips were starved for 1.5 hours in Hepes-buffered medium before incubation with 25 μM OHOA for 30 minutes at 37°C. The cells were then cooled down in cold Hepes-buffered medium for 15 minutes at 4°C to stop trafficking, and thereafter labeled with 10 μg/ml CD8 antibody (Sigma Aldrich) for 30 minutes at 4°C. After washing in cold PBS, the cells were incubated in warm Hepes-buffered medium at 37°C, in the presence or absence of OHOA, and chased for 8 or 16 minutes. The cells were then washed with PBS, fixed in 10% formalin (Sigma Aldrich), washed again and permeabilized with 0.1% Triton X-100 for 2 minutes at room temperature. The cells were washed again, followed by blocking in 5% FBS in PBS for 30 minutes. Finally the cells were incubated with Alexa555-anti-mouse secondary antibody (Jackson Laboratories) for 1 hour at room temperature, washed and mounted with ProLong Gold antifade mounting medium with DAPI (Molecular Probes). The cells were imaged as described above. Fiji software [57] was applied to quantify transport of CD8-CIMPR to the Golgi. After background subtraction, the GFP-GalT signal was used to create a Golgi mask, and the integrated signal intensity of CD8-CIMPR in the Golgi as well as in the whole cell was measured. Transport to the Golgi was then reported as the signal of CD8-CIMPR in the Golgi as percentage of total cellular CD8-CIMPR. At least 40 cells were analyzed per condition and time point, and the experiment was performed 4 times.

### Statistical analysis

Mean values ± standard error of the mean (SEM) were calculated for each condition. The statistical significance of the differences was determined by two-tailed Student's *t-test*, either paired or with unequal variances, as appropriate; *p < 0.05; **p < 0.01; ***p < 0.001.

## SUPPLEMENTARY MATERIALS FIGURES


