# Supplementary material for: The anti-tumor drug 2-hydroxyoleic acid (Minerval) stimulates signaling and retrograde transport
Source: Oncotarget. 2016 Nov 22;7(52):86871–88. doi: 10.18632/oncotarget.13508 (PMC5349960; doi:10.18632/oncotarget.13508)
Supplement: Supplementary file 1 [file oncotarget-07-86871-s001.pdf]

The anti-tumor drug 2-hydroxyoleic acid (Minerval) stimulates signaling and retrograde transport

Supplementary Materials

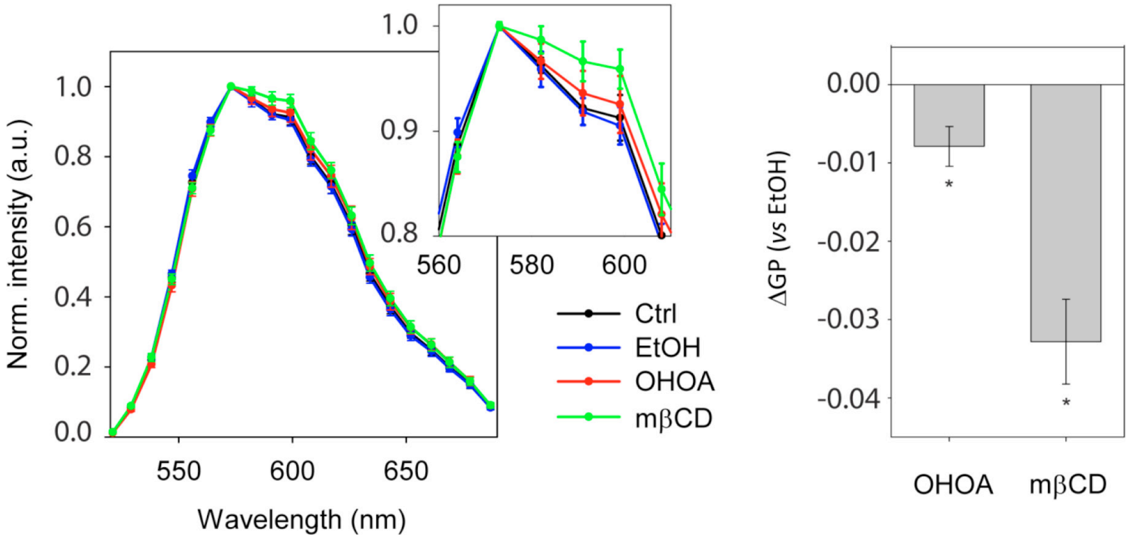

Supplementary Figure S1: OHOA treatment reduces plasma membrane lipid packing. HeLa cells were treated with 50 µM OHOA or 0.05% ethanol for 30 minutes, or with 5 mM mβCD for 1 hour, prior to staining with 10 nM NR12S. Spectral imaging was started 7 minutes after the addition of NR12S and continued for 10 minutes. The analysis of the spectra was performed on at least 20 manually selected regions of the plasma membrane for each condition. The graph to the left shows the spectral data from one independent experiment (± SD). The average GP value for each selection was quantified as described in Materials and Methods, and the bars to the right shows differences in GP values (mean ± SEM) between the EtOH treated samples and the OHOA and mβCD samples quantified from at least three independent experiments; *$p < 0.05$.

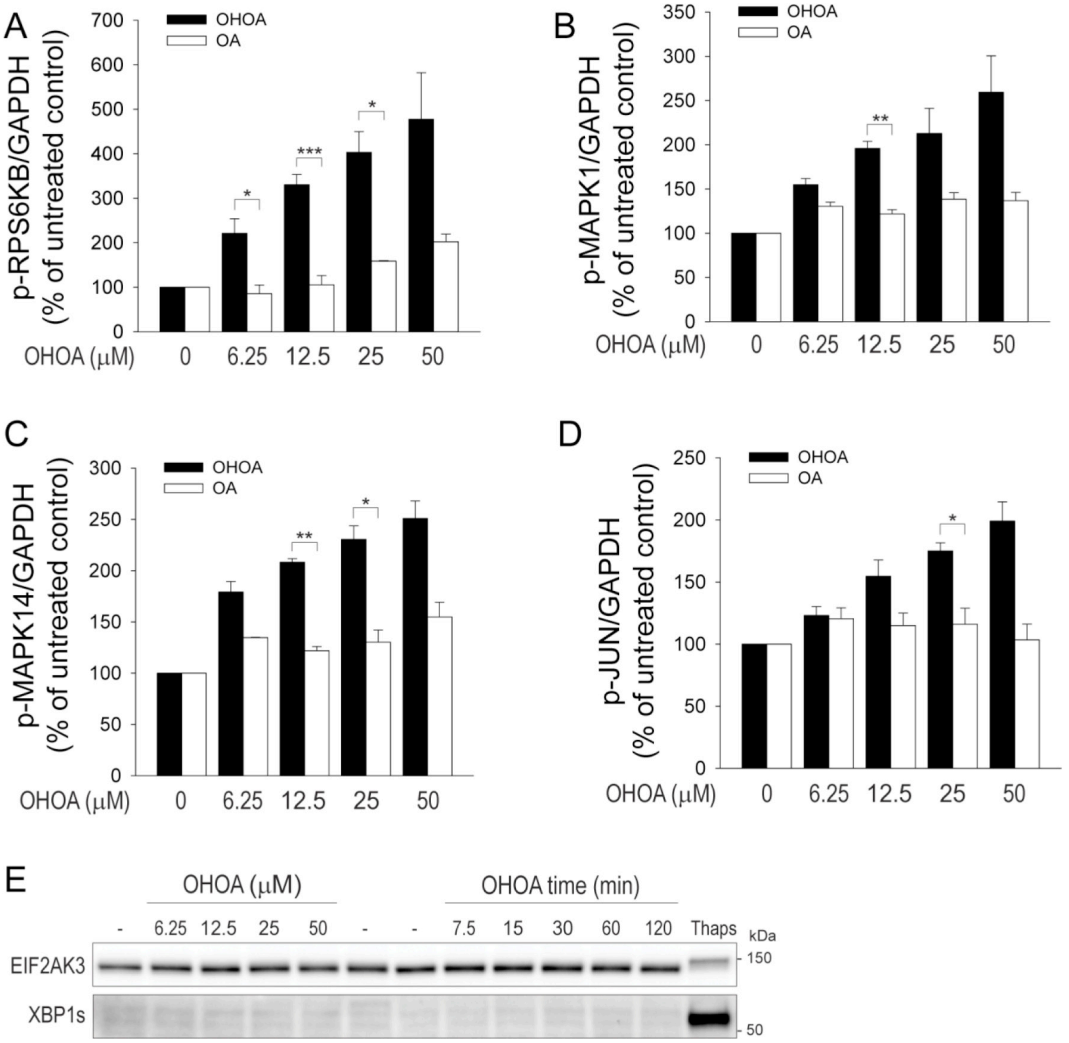

Supplementary Figure S2: OHOA stimulates cellular signaling, but not ER stress. (**A**–**D**). HeLa cells were treated with the indicated concentrations of OHOA or OA for 30 minutes and cell lysates were prepared for immunoblotting. The blots were probed with the indicated antibodies, and the signal intensities quantified. The relative levels of p-RPS6KB (A), p-MAPK1 (B), p-MAPK14 (C), and p-JUN (D) were normalized to GAPDH. All bars show mean values + SEM quantified from at least three independent experiments; $*p < 0.05$; $**p < 0.01$; $***p < 0.001$. (E) HeLa cells were treated with the indicated concentrations of OHOA for 30 minutes, or treated with 12.5 μM OHOA for the indicated time periods, and cell lysates were prepared for immunoblotting. Treatment with 100 nM thapsigargin for 3 hours was used as a positive control. The blots were probed with the indicated antibodies. ER stress is indicated by phosphorylation of EIF2AK3, detected as an upward shift, or detection of the alternatively spliced XBP1, termed XBP1s.

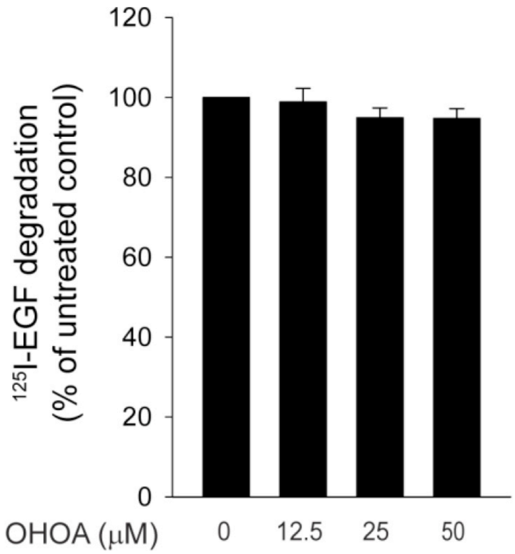

Supplementary Figure S3: OHOA does not increase EGF degradation. HeLa cells were treated with the indicated concentrations of OHOA for 30 minutes before addition of ^{125}I-EGF. EGF was internalized for 10 minutes, then the cells were washed, and EGF was allowed to degrade for 1 hour. Proteins were precipitated with TCA, and free- and protein-bound radioactivity was determined. EGF degradation was calculated as TCA-soluble radioactivity as percent of total radioactivity (TCA-soluble + insoluble). The bars show mean values + SEM quantified from at least 3 independent experiments.

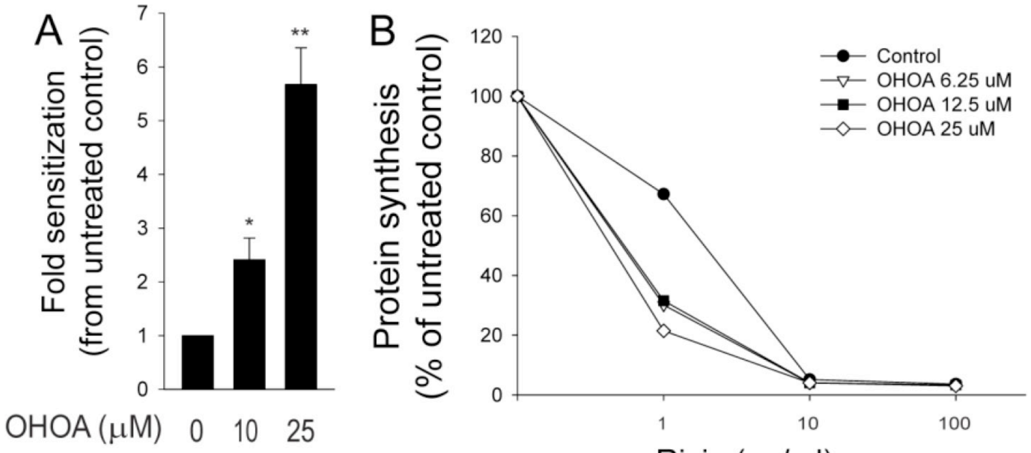

Supplementary Figure S4: OHOA stimulates ricin toxicity in HEp-2 and U2-OS cells. (**A**) HEp-2 cells were preincubated with the indicated concentrations of OHOA in leucine-free medium for 30 minutes, then increasing concentrations of ricin were added and the incubation continued for 3 hours. The protein synthesis was measured as described in Materials and Methods. The bars represent fold sensitization against ricin at 50% inhibition of protein synthesis and show mean values + SEM quantified from at least 3 independent experiments. (**B**) U2-OS cells were treated as in A and data from one representative experiment are shown.

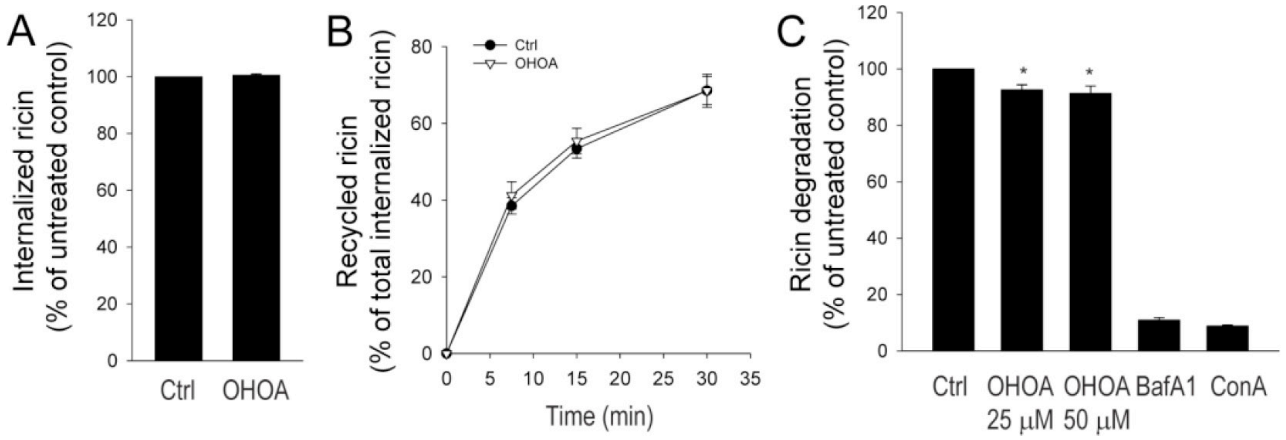

Supplementary Figure S5: OHOA does not alter ricin endocytosis, recycling or degradation. (**A**) HeLa cells were treated with 25 μM OHOA before 200 ng/ml ^{125}I-ricin was added and allowed to internalize for 20 minutes. The data from OHOA-treated cells was normalized to that of untreated control cells, and the bars show mean values ± SEM quantified from at least 3 independent experiments. (**B**) HeLa cells were treated with 25 μM OHOA before 200 ng/ml ^{125}I-ricin was added and allowed to internalize for 30 minutes. Surface-bound ricin was removed by lactose, and the recycled ricin was measured at the indicated time-points. The plots show mean values ± SEM quantified from at least 3 independent experiments. (**C**) HeLa cells were treated with 25 or 50 μM OHOA, 50 nM bafilomycin A1 (BafA1) or 50 nM concanamycin A (ConA) before 200 ng/ml ^{125}I-ricin was added and allowed to internalize for 20 minutes. Surface-bound ricin was removed by lactose, and the incubation continued for 2 hours. Proteins were precipitated with TCA and free- and protein-bound radioactivity was determined. Ricin degradation was calculated as TCA-soluble radioactivity as percent of total radioactivity (TCA-soluble + insoluble). The bars show mean values ± SEM quantified from at least 3 independent experiments; $*p < 0.05$.

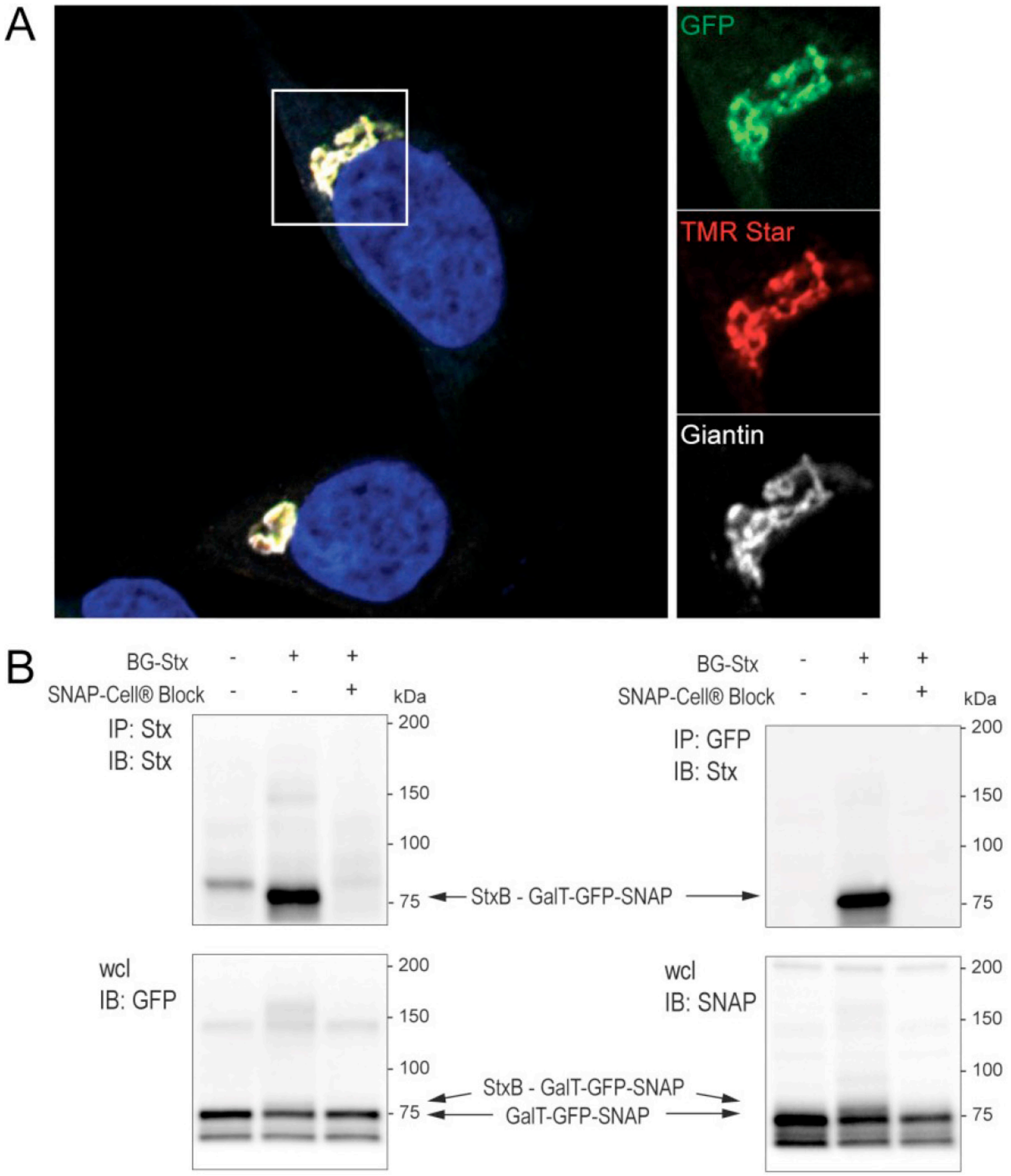

Supplementary Figure S6: Functional testing of the HeLa-GalT-GFP-SNAP cell line. (**A**) HeLa-GalT-GFP-SNAP cells were treated with the fluorescent benzylguanine (BG) derivative SNAP-Cell® TMR-Star according to the manufacturer's instructions. The cells were then fixed and prepared for immunofluorescence with anti-giantin antibodies. The staining shows that GalT-GFP-SNAP is functional and localized to the Golgi. (**B**) To verify that BG-labeled retrograde cargo is coupled to the SNAP-tag in this cell line, we chose Shiga toxin (Stx) as a model cargo, as a large fraction of the internalized toxin molecules is routed into the retrograde pathway. HeLa-GalT-GFP-SNAP cells were treated with 500 ng/ml BG-labeled Stx for 2 hours in the absence or presence of SNAP-Cell® Block, which inhibits the SNAP-tag coupling reaction. The SNAP-tagged toxin was then immunoprecipitated with either Stx antibodies or GFP-trap® (ChromoTek), and detected by immunoblotting with anti-Stx antibodies. The specific band at appox. 80 kDa corresponds to GalT-GFP-SNAP coupled to one Stx B-subunit of approx. 5 kDa. The same band is also weakly detectable in the whole cell lysates (wcl, lower panels) right above the band corresponding to the total GalT-GFP-SNAP migrating at approx. 75 kDa, especially when detected by anti-SNAP antibodies.

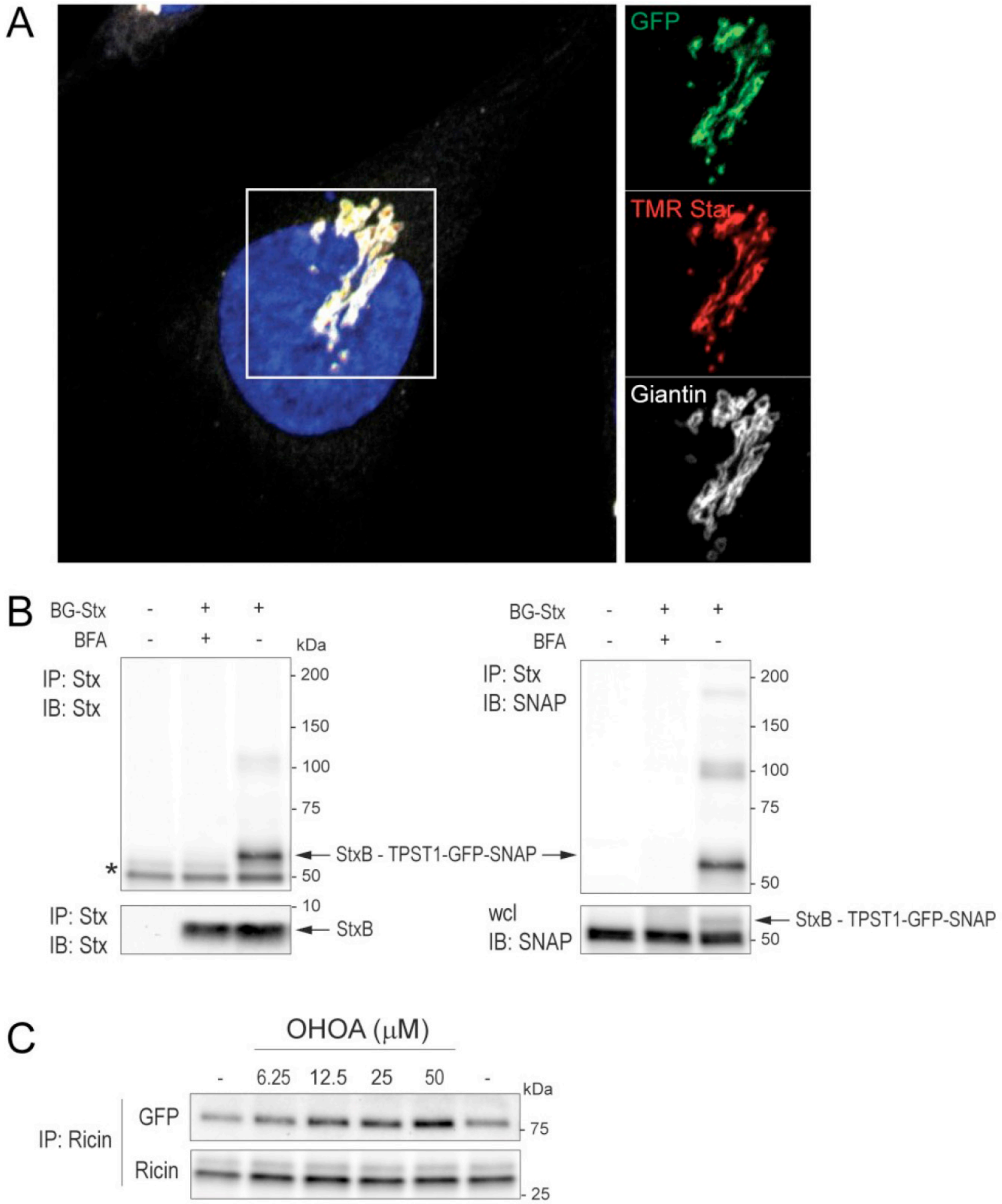

Supplementary Figure S7: Functional testing of the HeLa-TPST1-GFP-SNAP cell line. (**A**) HeLa-TPST1-GFP-SNAP cells were treated with the fluorescent benzylguanine (BG) derivative SNAP-Cell® TMR-Star according to the manufacturer's instructions. The cells were then fixed and prepared for immunofluorescence with anti-giantin antibodies. The staining shows that TPST1-GFP-SNAP is functional and localized to the Golgi. (**B**) To verify that retrograde cargo is able to couple to the SNAP-tag in HeLa-TPST1-GFP-SNAP cells, they were treated with 500 ng/ml BG-labeled Stx for 2 hours in the absence or presence of 5 µg/ml brefeldin A (BFA), which disrupts the Golgi complex. The SNAP-tagged toxin was then immunoprecipitated with Stx antibodies, and detected by immunoblotting with the indicated antibodies. The specific band at 55–60 kDa corresponds to TPST1-GFP-SNAP coupled to one StxB subunit of approx. 5 kDa. The slight shift generated by the coupling to StxB is also weakly detectable in the whole cell lysates (wcl, lower right panel). The band marked with an asterix represents the IgG heavy chain. (**C**) HeLa-TPST1-GFP-SNAP cells were treated with the indicated concentrations of OHOA and assayed for Golgi transport by the SNAP-tag assay as described in Materials and Methods. Representative immunoblots of total- and SNAP-tagged ricin are shown.

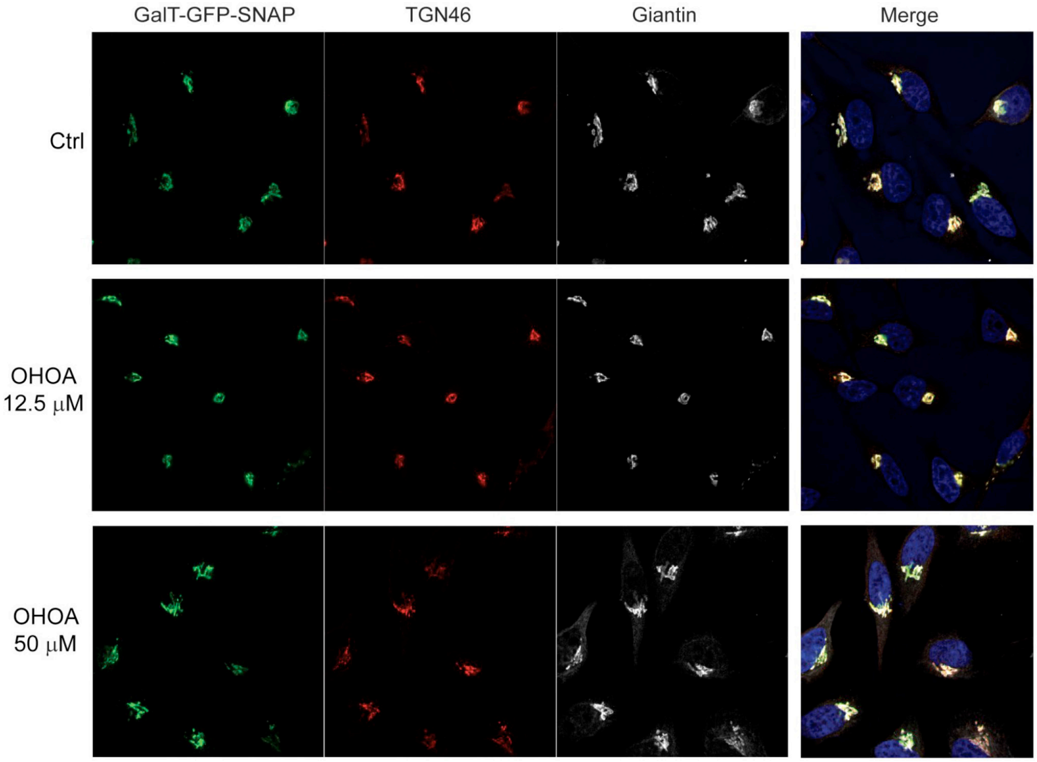

Supplementary Figure S8: OHOA does not alter the structure of the Golgi complex. HeLa-GalT-GFP-SNAP cells were treated with the indicated concentrations of OHOA for 1 hour, fixed and prepared for immunofluorescence with the indicated antibodies. No major alterations in Golgi structure were detectable.

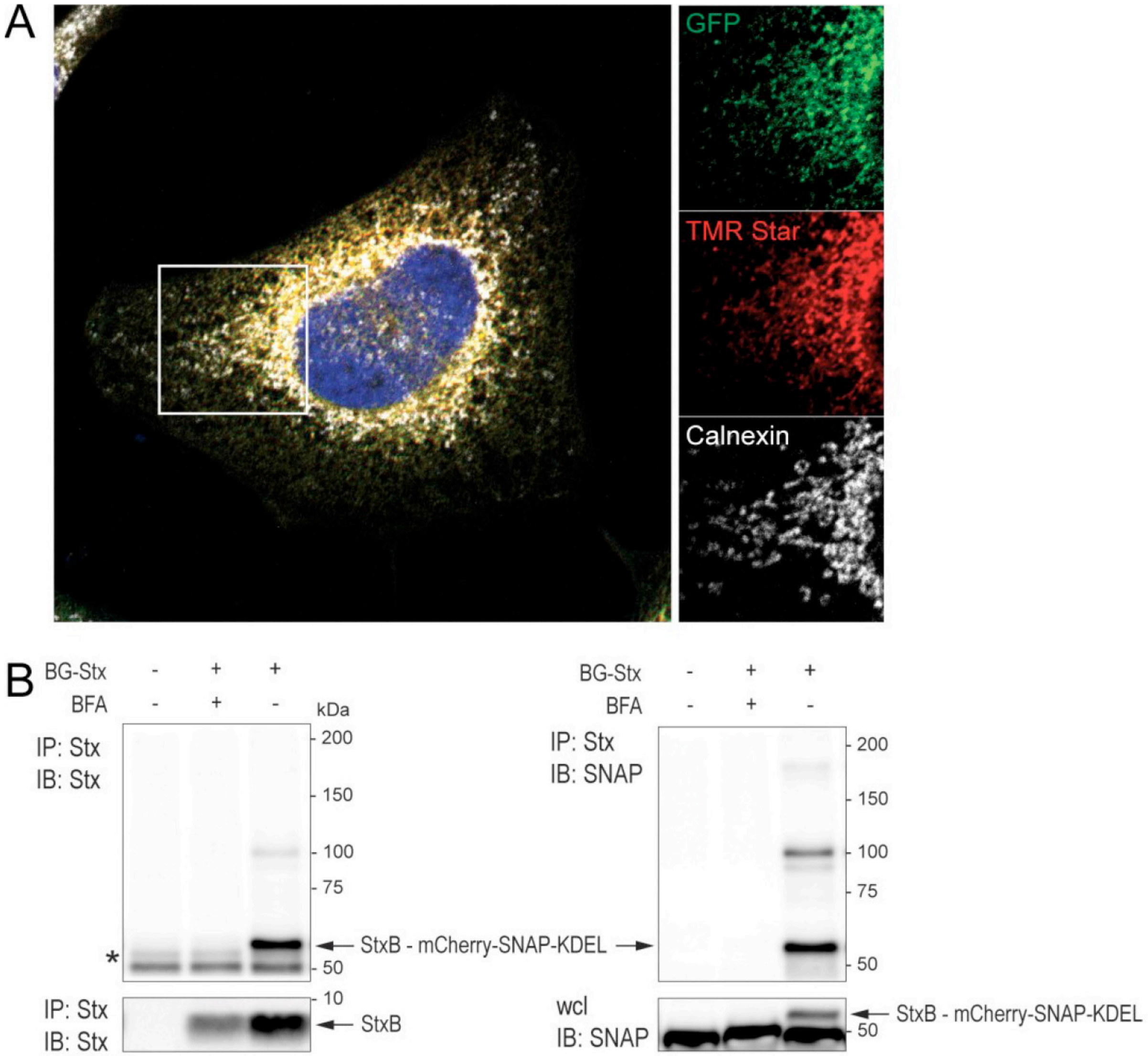

Supplementary Figure S9: Verification of the HeLa-ER-SNAP-KDEL cell lines. (**A**) HeLa-ER-GFP-SNAP-KDEL cells were treated with the fluorescent benzylguanine (BG) derivative SNAP-Cell® TMR-Star according to the manufacturer's instructions. The cells were then fixed and prepared for immunofluorescence with anti-calnexin antibodies. The staining shows that ER-GFP-SNAP is functional and localized to the ER. When the GFP of the ER-GFP-SNAP-KDEL cell line was replaced with mCherry, an identical ER-localization of the SNAP-tag was detectable (data not shown). (**B**) To verify that retrograde cargo is able to couple to the SNAP-tag in HeLa-ER-mCherry-SNAP cells, they were treated with 500 ng/ml BG-labeled Stx for 2 hours in the absence or presence of 5 µg/ml brefeldin A (BFA), which disrupts the Golgi complex. The SNAP-tagged toxin was then immunoprecipitated with Stx antibodies, and detected by immunoblotting with the indicated antibodies. The specific band at 55–60 kDa corresponds to ER-GFP-SNAP-KDEL coupled to one StxB subunit of approx. 5 kDa. The slight shift generated by the coupling to StxB is also weakly detectable in the whole cell lysates (wcl, lower right panel). The band marked with an asterix represents the IgG heavy chain.

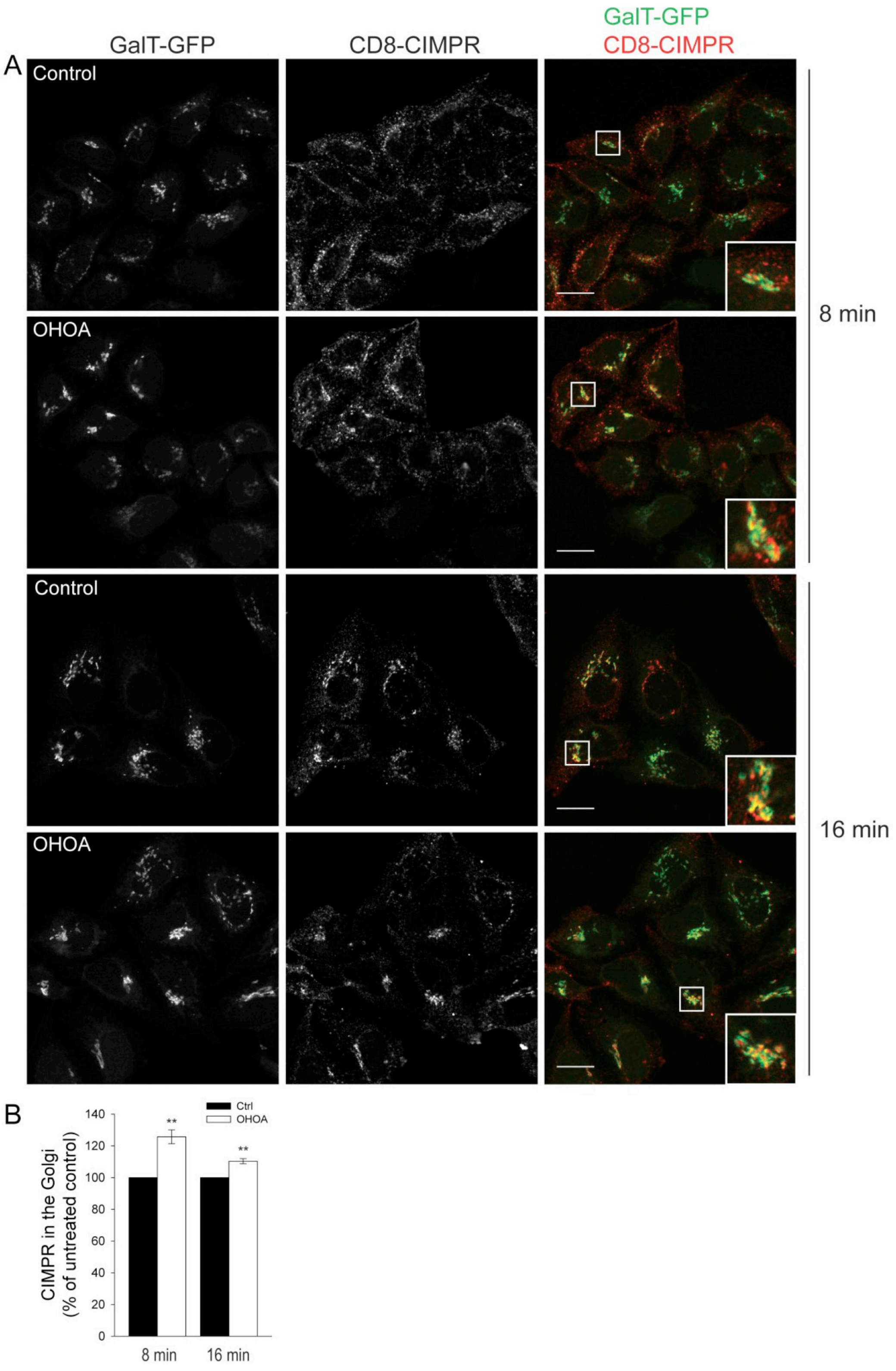

Supplementary Figure S10: OHOA stimulates Golgi transport of CIMPR. HeLa CD8-CIMPR/GalT-GFP cells were treated with 25 µM OHOA for 30 minutes before labeling with anti-CD8 antibodies and detection of CD8-CIMPR transport to the Golgi as described in Materials and Methods. (**A**) Representative images from the 8- and 16 minutes timepoints. Scale bar; 10 µm. (**B**) The bars show mean values ± SEM quantified from at least 3 independent experiments. $**p < 0.01$ compared to untreated control.

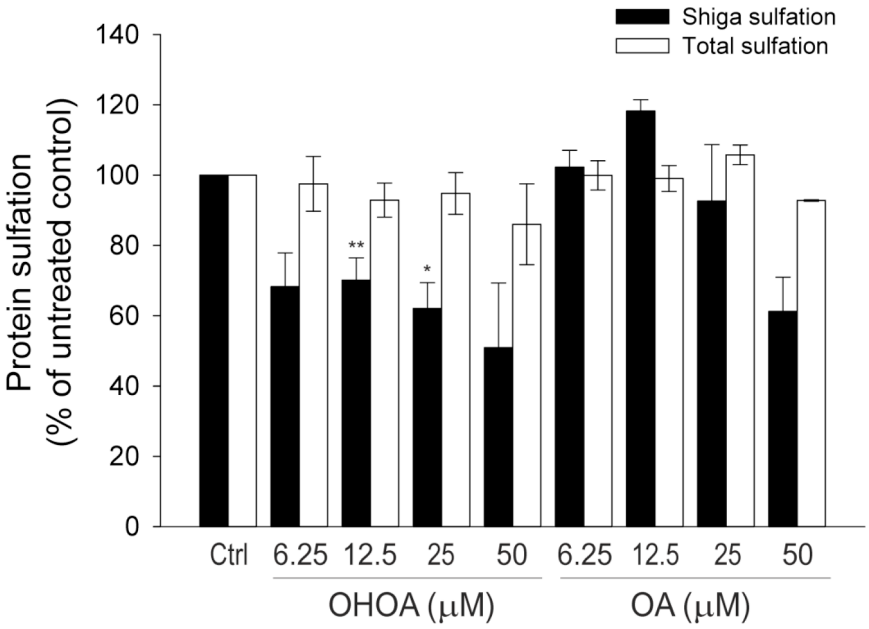

Supplementary Figure S11: OHOA reduces Stx retrograde transport. HeLa cells were treated with the indicated concentrations of OHOA or OA and assayed for Stx sulfation as described in Materials and Methods. The bars show mean values ± SEM quantified from at least 3 independent experiments; $*p < 0.05$; $**p < 0.01$ compared to untreated control.

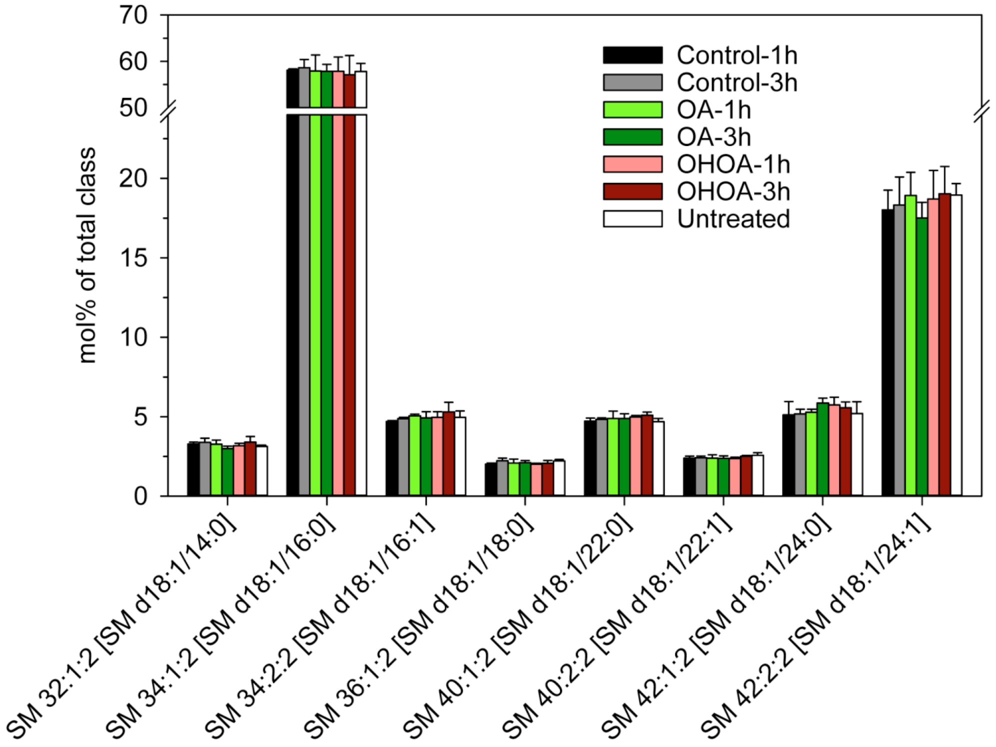

Supplementary Figure S12: Lipidomics of HeLa cells treated with OHOA or OA. HeLa cells were treated with 12.5 µM OHOA or OA for 1 or 3 hours before the cells were subjected to lipidomics analysis as detailed in Materials and Methods. The bars show the mol% of the SM lipid species as percentage of total SM; mean values + SEM quantified from 3 independent experiments.

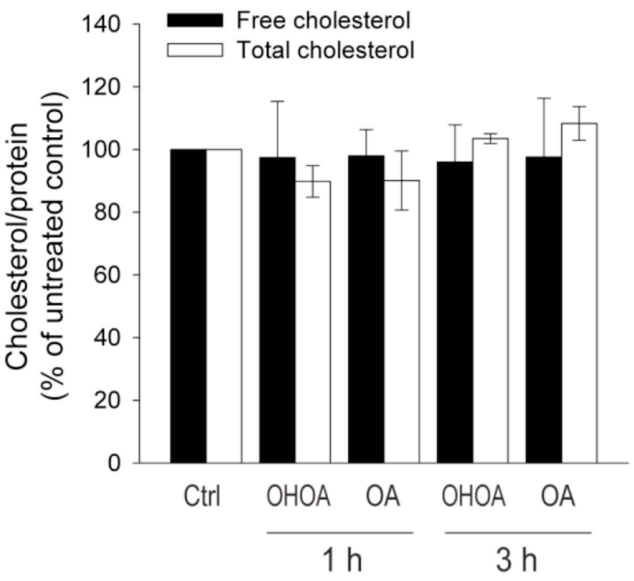

Supplementary Figure S13: The cholesterol levels are unaltered in HeLa cells treated with OHOA or OA. HeLa cells were treated with 12.5 µM OHOA or OA for 1 or 3 hours before the cells were subjected to cholesterol analysis as detailed in Materials and Methods. The bars show cholesterol levels normalized to protein amount as percentage of untreated control; mean values ± deviation from mean quantified from 2 independent experiments.

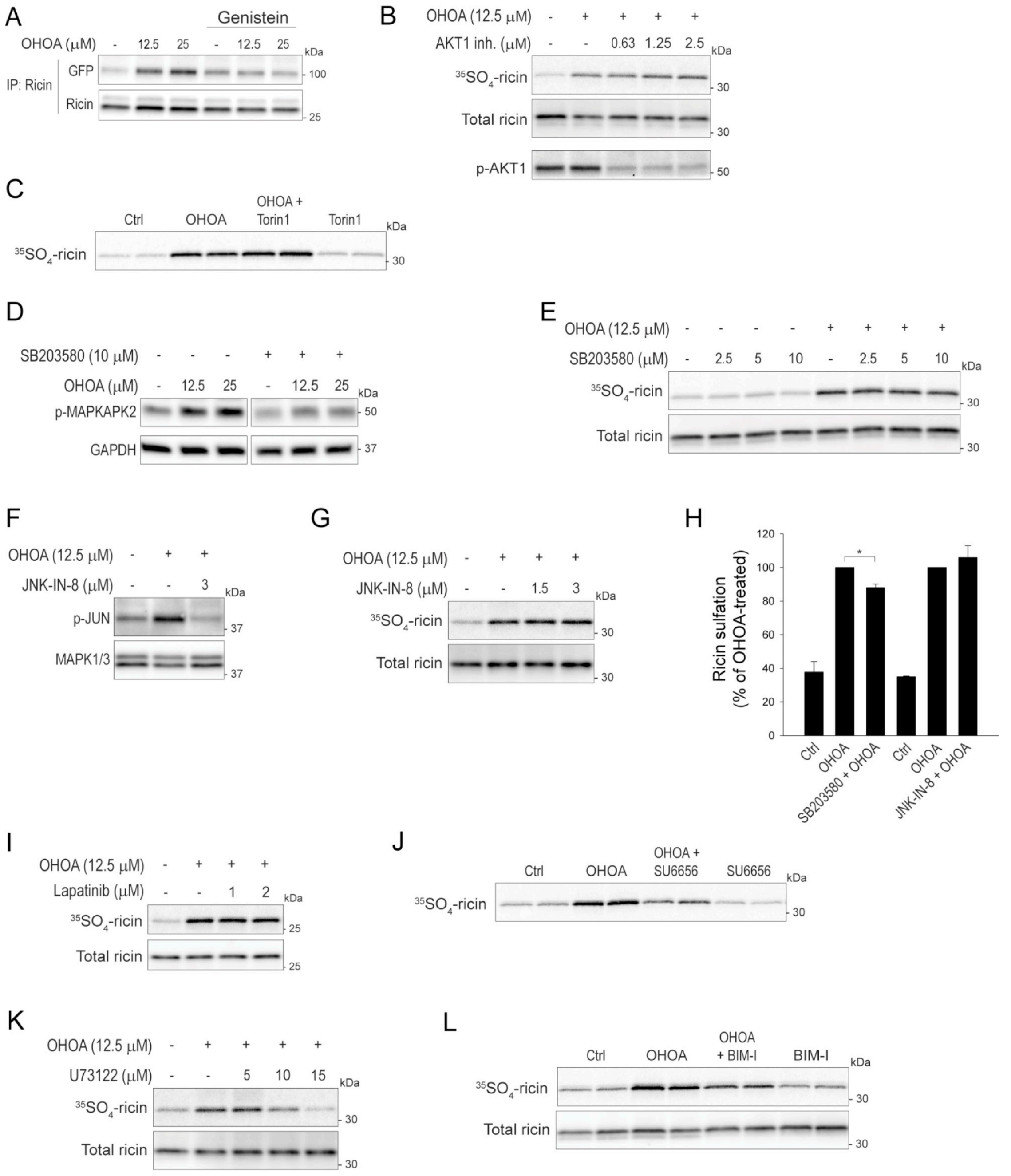

Supplementary Figure 14: OHOA-stimulated retrograde transport of ricin is inhibited by genistein, SU6656, U73122 and BIM-I. (**A**) HeLa-GalT-GFP-SNAP cells were treated with the indicated concentrations of OHOA in the presence or absence of 25 μg/ml genistein, and assayed for Golgi transport of ricin by the SNAP-tag assay as described in Materials and Methods. Representative immunoblots of total- and SNAP-tagged ricin are shown. (**B**) HeLa cells were treated with the indicated concentrations of AKT1 inhibitor VIII and OHOA and ricin sulfation was determined as described in Materials and Methods. The basal phosphorylation of AKT1 in the cell lysate was determined by immunoblotting. (**C**) HeLa cells were treated with 12.5 μM OHOA in the presence or absence of 50 nM Torin1 and ricin sulfation was determined as above. (**D**) HeLa cells were treated with the indicated concentrations of OHOA for 1 hour in the presence or absence of 10 μM SB203580. The phosphorylation-status of the p38 downstream target MAPKAPK2 was determined by immunoblotting. (**E**) HeLa cells were treated with 12.5 μM OHOA and the indicated concentrations of SB203580, and ricin sulfation was determined as above. (**F**) HeLa cells were treated with 12.5 μM OHOA for 1 hour in the presence or absence of 3 μM JNK-IN-8. The phosphorylation-status of the MAPK8 downstream target JUN was determined by immunoblotting. (**G**) HeLa cells were treated with 12.5 μM OHOA and the indicated concentrations of JNK-IN-8, and ricin sulfation was determined as above. (**H**) Quantifications of the data obtained in E and G. The bars show mean values ± SEM quantified from at least 3 independent experiments. *$p < 0.05$. (**I**) HeLa cells were treated with 12.5 μM OHOA and the indicated concentrations of lapatinib, and ricin sulfation was determined as above. (**J**) HeLa cells were treated with 12.5 μM OHOA in the presence or absence of 5 μM SU6656 and ricin sulfation was determined as above. (**K**) HeLa cells were treated with 12.5 μM OHOA and the indicated concentrations of U73122, and ricin sulfation was determined as above. (**L**) HeLa cells were treated with 12.5 μM OHOA in the presence or absence of 10 μM BIM-I and ricin sulfation was determined as above.

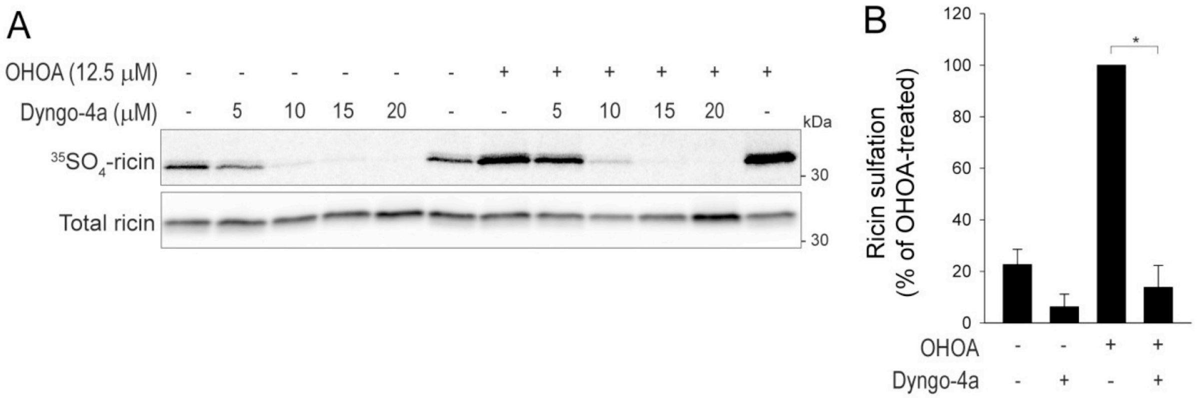

Supplementary Figure S15: Ricin retrograde transport is blocked by dynamin inhibitor. (**A**) HeLa cells were treated with the indicated concentrations of Dyngo-4a and 12.5 μM OHOA and ricin sulfation was determined as described in Materials and Methods. A representative sulfation autoradiograph with the corresponding immunoblot is shown. (**B**) Quantification of the data obtained in A. The bars show mean values ± SEM quantified from at least 3 independent experiments; $*p < 0.05$.

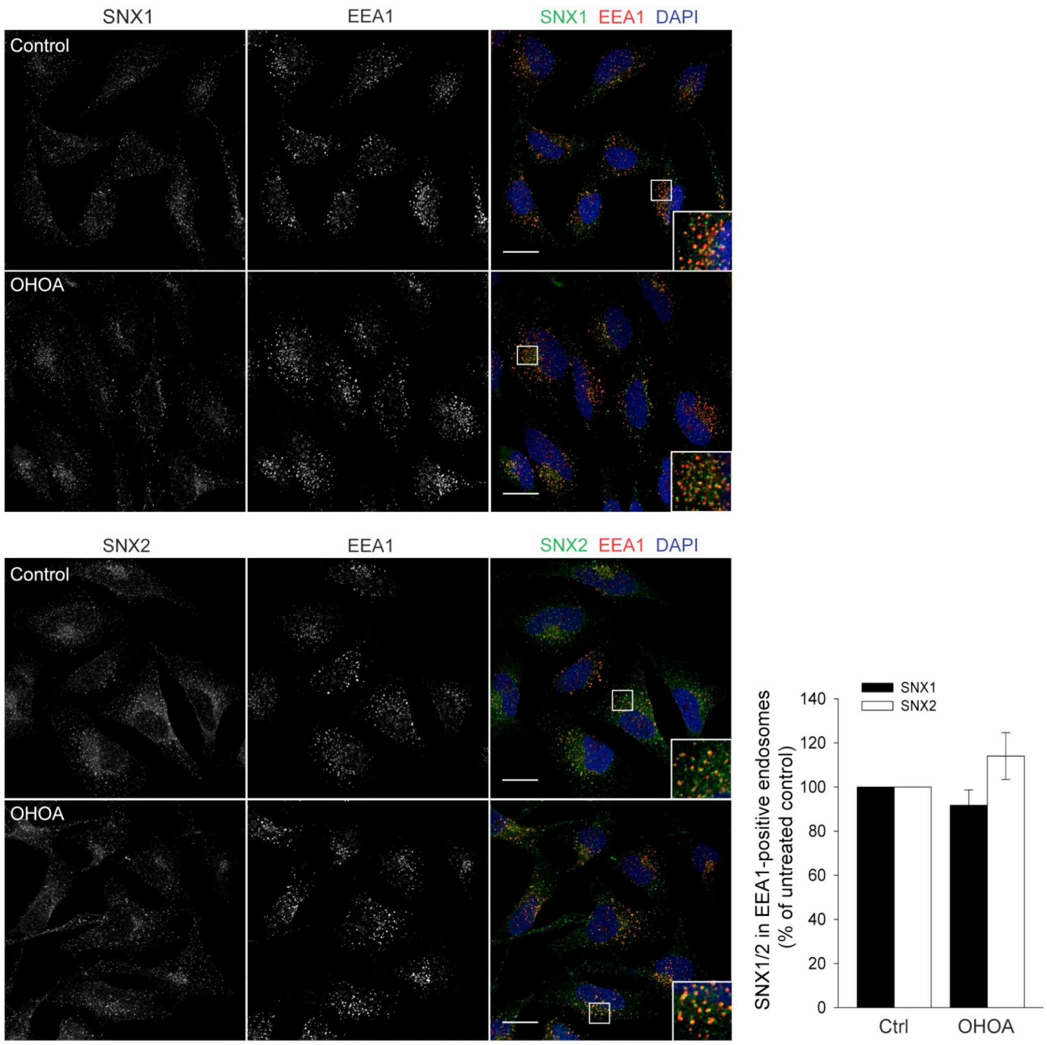

Supplementary Figure S16: OHOA does not alter the endosomal localization of SNX1 or SNX2. HeLa cells were treated with 25 μM OHOA for 30 minutes before fixation and staining with the indicated antibodies as described in Materials and Methods. Representative images are shown. Scale bar; 20 μm. Right panel: Quantification of SNX1 or SNX2 staining within the EEA1-positive endosome mask as compared to the total cellular staining of these markers. The data are presented as percent of untreated control values and the bars show mean values ± SEM quantified from at least 3 independent experiments.

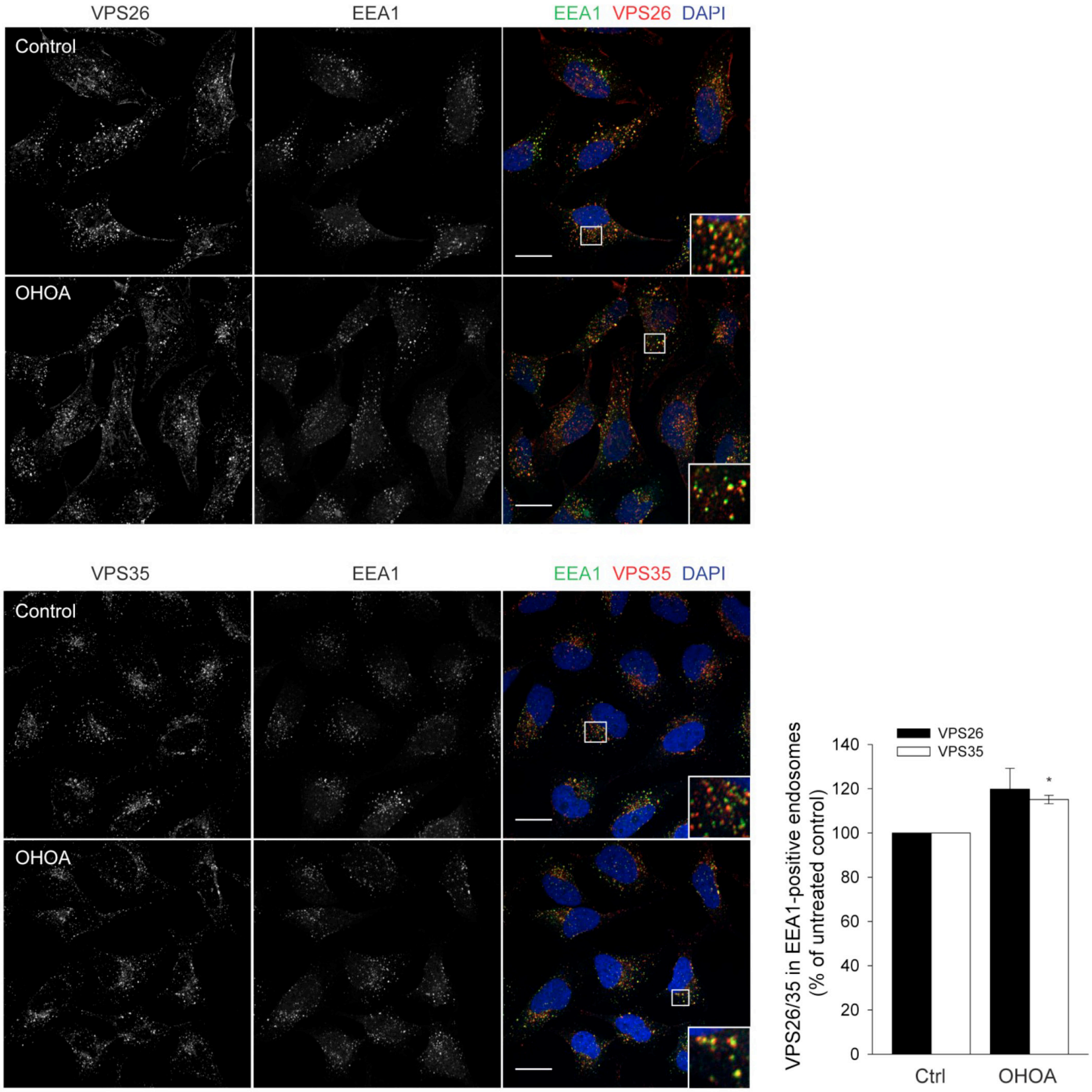

Supplementary Figure S17: OHOA increases the endosomal localization of VPS35. HeLa cells were treated with 25 µM OHOA for 30 minutes before fixation and staining with the indicated antibodies as described in Materials and Methods. Representative images are shown. Scale bar; 20 µm. Right panel: Quantification of VPS26 or VPS35 staining within the EEA1-positive endosome mask as compared to the total cellular staining of these markers. The data are presented as percent of untreated control values and the bars show mean values ± SEM quantified from at least 3 independent experiments. *$p < 0.05$.
